# An experiment-informed signal transduction model for the role of the *Staphylococcus aureus* MecR1 protein in β-lactam resistance

**DOI:** 10.1038/s41598-019-55923-z

**Published:** 2019-12-20

**Authors:** Bruno S. Belluzo, Luciano A. Abriata, Estefanía Giannini, Damila Mihovilcevic, Matteo Dal Peraro, Leticia I. Llarrull

**Affiliations:** 1 Instituto de Biología Molecular y Celular de Rosario (IBR, CONICET-UNR), Predio CONICET Rosario, 27 de Febrero 210 bis, 2000 Rosario, Argentina; 2 Laboratory for Biomolecular Modeling - École Polytechnique Fédérale de Lausanne and Swiss Institute of Bioinformatics, CH-1015 Lausanne, Switzerland; 3 Área Biofísica, Facultad de Ciencias Bioquímicas y Farmacéuticas, Universidad Nacional de Rosario, Suipacha 570, 2000 Rosario, Argentina

**Keywords:** Proteases, Computational models, Antimicrobial resistance

## Abstract

The treatment of hospital- and community-associated infections by methicillin-resistant *Staphylococcus aureus* (MRSA) is a perpetual challenge. This Gram-positive bacterium is resistant specifically to β-lactam antibiotics, and generally to many other antibacterial agents. Its resistance mechanisms to β-lactam antibiotics are activated only when the bacterium encounters a β-lactam. This activation is regulated by the transmembrane sensor/signal transducer proteins BlaR1 and MecR1. Neither the transmembrane/metalloprotease domain, nor the complete MecR1 and BlaR1 proteins, are isolatable for mechanistic study. Here we propose a model for full-length MecR1 based on homology modeling, residue coevolution data, a new extensive experimental mapping of transmembrane topology, partial structures, molecular simulations, and available NMR data. Our model defines the metalloprotease domain as a hydrophilic transmembrane chamber effectively sealed by the apo-sensor domain. It proposes that the amphipathic helices inserted into the gluzincin domain constitute the route for transmission of the β-lactam-binding event in the extracellular sensor domain, to the intracellular and membrane-embedded zinc-containing active site. From here, we discuss possible routes for subsequent activation of proteolytic action. This study provides the first coherent model of the structure of MecR1, opening routes for future functional investigations on how β-lactam binding culminates in the proteolytic degradation of MecI.

## Introduction

Methicillin-resistant *Staphylococcus aureus* (MRSA) is a huge challenge with respect to the control of hospital- and community-associated infection^1–7^. This bacterium is generally resistant to several antibacterial agents, and especially resistant to the β-lactam antibiotics^8^. Few new antibacterials are in advanced stages of clinical evaluation for the treatment of MRSA infection^9–13^ and resistant MRSA strains evolve rapidly^14–16^. Resistance to β-lactam antibiotics in MRSA is inducible^17,18^ and is the result of the expression of two resistance enzymes. One is the PC1 β-lactamase (BlaZ)^19^ and the second is the PBP2a transpeptidase. The causative event for the bactericidal mechanism of the β-lactams is inactivation of the critical PBP-catalyzed crosslinking of the peptidoglycan cell wall of the bacterium. In contrast to the other PBPs of *S*. *aureus*, PBP2a has low affinity for almost all β-lactam antibiotics, and hence is not inhibited by the concentrations of β-lactams that are achievable clinically^20^. The genes that code for these two resistance determinants are part of the *bla* and *mec* operons, respectively^21,22^. These operons also include the genes for a transmembrane sensor/transducer protein (BlaR1—β-lactam antibiotic receptor protein—and MecR1—methicillin receptor protein—, respectively) and a DNA-binding protein (BlaI and MecI, respectively). The *mec* operon also includes an additional protein, MecR2, which is an antirepressor^23^. The regulation of the expression of these resistance determinants by BlaR1 and MecR1 have strong mechanistic parallel: they both detect the presence of β-lactam antibiotics in the milieu using an extracellular sensor domain, by catalysis of an irreversible acylation of an active site serine by the β-lactam. This acylation is transduced to the cytoplasmic domain as a structural reorganization of the entire protein^24,25^. Neither the nature of this structural reorganization, nor its connection to control of protein expression, is known. The presence of an HEXXH motif in the cytoplasmic domain—a motif that is characteristic of a metalloproteinase^26^—is the center of current hypotheses. The two conserved His residues of this motif are suggested to provide two of the ligands to an active site Zn(II) atom^27^. Studies on BlaR1 from *Bacillus licheniformis* identified a glutamic acid as the third zinc-binding ligand^28^. If correct, these structural features place the metalloprotease domain in the gluzincin family^29^. Although changes in the secondary structure of the *S. aureus* proteins are seen using circular dichroism (CD) and Fourier-transform Infrared spectroscopy (FTIR) assays^24,30^, these changes are not evident in the X-Ray structures of the solubilized, apo-sensor domain and β-lactam-bound sensor domain^31,32^. BlaR1 has proven metalloprotease activity, as it catalyzes proteolysis of BlaI and/or undergoes autoproteolysis^19,27^. BlaI binds to the operator region of the *blaR* operon, and when bound represses transcription to a low basal level^33^. Loss of BlaI, such as occurs by proteolysis, results in BlaZ expression resulting in β-lactam resistance^27^. In contrast, no direct evidence implicates MecR1 proteolysis of MecI, although this event is credible. Full activation of the *mec* operon requires the presence of MecR2. MecR2 binds to MecI and presumably promotes MecI degradation mediated by native cytoplasmatic proteases^23^. Hence, MecR2 interferes with interaction of MecI with the *mecA* promoter.

Our understanding of the activation of BlaR1 and MecR1 by β-lactams is limited by the absence of X-Ray structures of the full proteins. We report a combined computational and experimental study that unveils the architecture of MecR1. Our model proposes a hydrophilic transmembrane chamber for the metalloprotease domain, wherein this domain interacts both with the sensor domain through an amphipathic helix and a “reentrant” helix. This reentrant helix is poised to propagate structural perturbation to the zinc site. This model allows us to formulate a proposal for the molecular route by which β-lactam acylation of the extracellular domain results in the activation of the cytoplasmic metalloprotease domain.

## Results

### Coevolution-based methods and homology modeling independently predict a gluzincin-like active site embedded in a transmembrane supermotif

**Figure 1:**
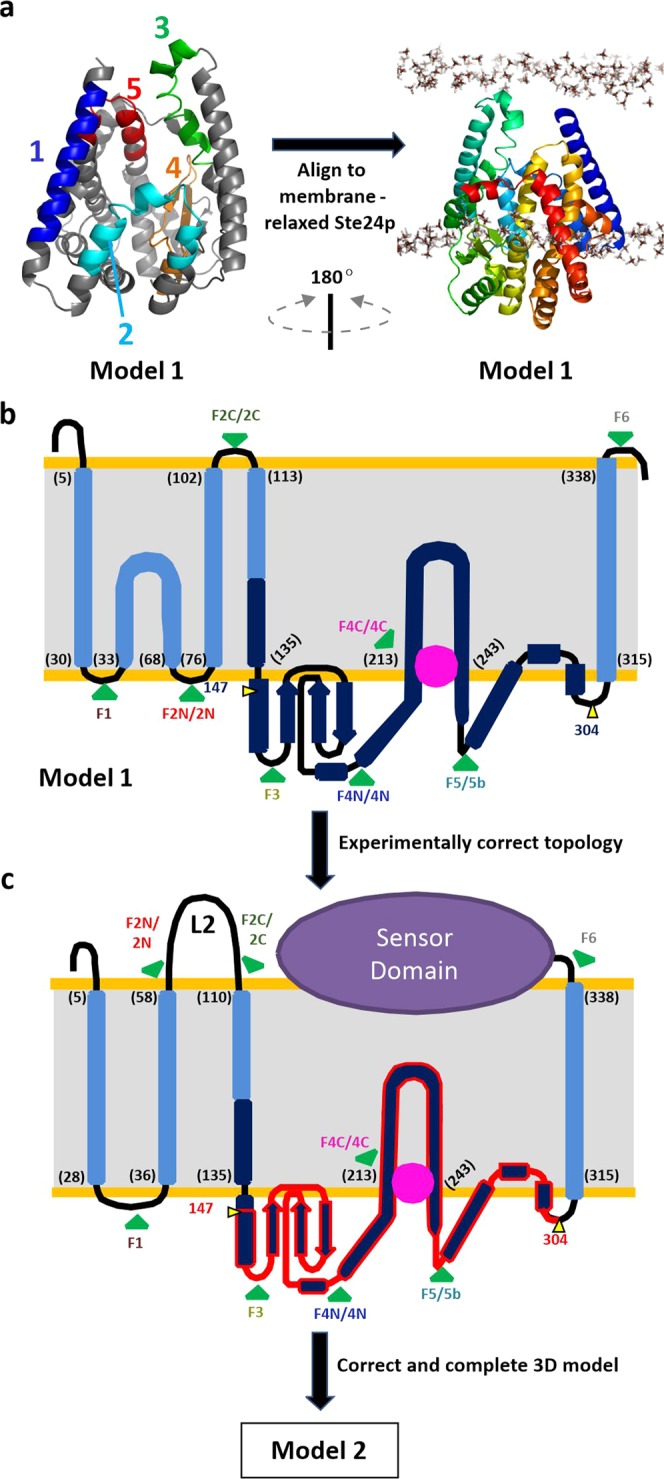
(**a**) Robetta model (Model 1) of residues 1-340 of the metalloprotease domain of MecR1 (cartoon). The left half maps each of the five transmembrane helices predicted from sequence (Table 1), colored from blue (N-terminal) to red (C-terminal). The right half depicts this model aligned to a membrane-relaxed structure of the Ste24p membrane metalloprotease (see Fig. S2). (**b**) Flat scheme showing the overall topology of the metalloprotease domain of MecR1 according to Model 1; light and dark blue for the whole homology model, dark blue for the portion backed up by coevolution data. (**c**) Flat scheme showing the overall topology of full-length MecR1 according to Model 2, which contemplates the results from the experimental mapping of the transmembrane topology. The gluzincin core of MecR1 (MecR1^GLZ^, residues 147–304) is shown in dark blue with red border. In (**b**,**c**), the numbers between brackets indicate the amino acid residue where each membrane-embedded helix is proposed to start or finish; the pink sphere approximates the position of the zinc ion; the green arrows labelled F1, F2N/2N, F2C/2C, F3, F4N/4N, F4C/4C, F5/5b and F6 indicate the positions where either eGFP was fused or a TEV recognition site was inserted for the experimental topology mapping.

The sensor proteins BlaR1 and MecR1 present an N-terminal metalloprotease domain (residues 1–338) and a C-terminal sensor domain (339–585)^24,25^. Analysis of MecR1 from *S. aureus* N315 (NRS70) using different algorithms predict three transmembrane (TM) segments in the N-terminal region (residues 1–130; Table 1), and most algorithms additionally predict a fourth TM helix before the C-terminal sensor domain (around residues 316–338). Some algorithms identify two additional low-score hydrophobic segments in the region that has historically been considered the cytoplasmic domain (residues 126–315). BLAST searches of the MecR1 metalloprotease domain against the Protein Data Bank returned few entries, all of low similarity. Thus, the homology modeling servers Robetta, I-TASSER, and Swiss-Model find only four proteins as templates for molecular modeling, covering only ~40–60% of the query sequences and with an identity of only 8.4 to 17.7%. These structures (PDB IDs 4AW6, 4IL3, 2YPT, and 5SYT) are gluzincin motifs embedded in helical transmembrane domains that define an internal hydrophilic chamber^34,35^. The gluzincin fold was consistently transferred by the four programs to the metalloprotease domain (residues 130–300) of MecR1. Coevolution-based modeling with EVFold^36^ independently predicted a similar structure for region 130–300, thus supporting the models built from distant homology. In particular, one homology model returned by Robetta (Model 1) matched very well the coevolution data (Figs. 1a and S1). The convergent consensus emerged from these independent modeling efforts was robust enough to test the resulting model experimentally. Hitherto, our homology- and coevolution-based models all indicated that (i) the gluzincin domain is embedded in a transmembrane helical domain similar to the gluzincin domain of the Ste24p and ZMPSTE24 proteases, and (ii) that one of the potential helices predicted by TopPred, HMMTOP and TMpred (around 172–193, the fourth helix) folds as a small helix and three β-strands as part of the gluzincin domain. We exploited these similarities to the Ste24p and ZMPSTE24 proteases to further model residues 1–330.

**Table 1:** Topology prediction of MecR1 from *Staphylococcus aureus* NRS70.

		TopPred	HMMTOP	TMHMM	TMpred	DAS
MecR1 *Staphylococcus aureus* NRS70						
TM regions	1	3–23	6–23	5–27	5–28	7–27
	2	37–57	40–57	40–62	37–55	39–54
	3	105–125	105–122	103–125	105–125	106–125
	4	172–192	162–179	—	173–193	—
	5	216–236	—	—	214–236	220–228
	6	316–336	—	316–338	316–334	319–330

Five distinct algorithms were used: TopPred, HMMTOP, TMHMM, TMpred and DAS, all available in www.expasy.org. Six transmembrane (TM) helical regions were found, with different confidence score levels regarding each algorithm. The numbers in black show the amino acid position in which the TM is located. Numbers in red correspond to TM helical regions predicted with a low score (below the cutoff stipulated in each algorithm).

### The metalloprotease domain of MecR1 defines a membrane-embedded hydrophilic chamber

The top two templates used by Robetta for the 1–340 sequence of MecR1 were PDB IDs 4AW6 and 2YPT, two X-ray structures of the human nuclear membrane zinc metalloprotease ZMPSTE24 (FACE1)^34^. In ZMPSTE24, seven long transmembrane helices (including two of the gluzincin domain) and one amphipathic helix close access to the hydrophilic chamber on the outer membrane side. We evaluated how this protein was accommodated in a membrane. Using coarse-grained and atomistic molecular dynamics simulations, we computationally relaxed the structure of the homologous protein Ste24p from *Saccharomyces mikatae* (PDB ID 4IL3, a higher resolution structure with fewer missing residues compared to the alternative structures)^35^ in a POPE membrane. This relaxation led to a match of the hydrophobic surface of Ste24p with the hydrophobic interior of the membrane, with the hydrophilic protein surface protruding on both sides of the membrane (Fig. S2). Fitting Model 1 for the metalloprotease domain of MecR1 to our membrane-equilibrated Ste24p (Fig. 1a) leads to two key observations. First, the topology of its hydrophobic segments is partially inconsistent with sequence-based predictions. This conclusion centers on the presence of two reentrant helices (2^nd^ and 5^th^ as predicted from the sequence) whose two termini are located in the cytosolic side and, as pointed out above, on the presence of one predicted helix (4^th^) that folds as part of the gluzincin domain inside the chamber (as is seen in the Ste24p protease). Second, the open side of the chamber of MecR1 would not reach the outer polar region of the membrane. As a result, several hydrophilic residues of the transmembrane helices and the interior of the chamber are directly exposed to the hydrophobic portion of the membrane (Fig. 1a). This unlikely structure for the transmembrane domain is a consequence of MecR1 having fewer helices, which are shorter and hence would not be able to span the membrane bilayer.

### Mapping of the topology of the metalloprotease domain of MecR1

**Figure 2:**
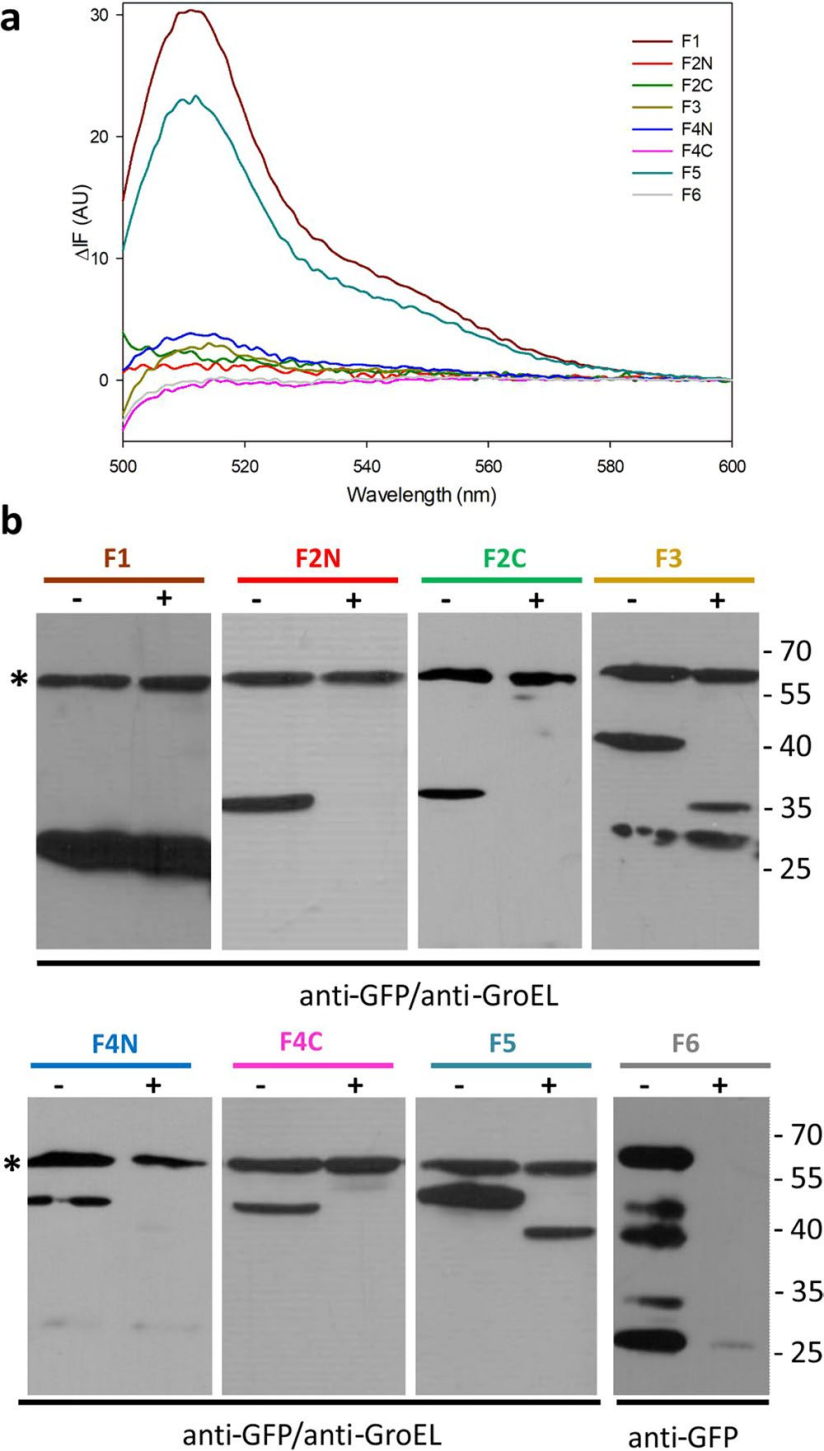
Topology mapping experiments of MecR1 using truncated fusions to eGFP. (**a**) Fluorescence emission spectra of each MecR1-eGFP fusion in membrane preparations, after subtraction of the spectra of the membrane proteins from cells harboring the empty vector. (**b**) Proteinase K protection assay of the MecR1-eGFP fusion proteins. Spheroplasts were incubated in the presence (+) or absence (–) of Proteinase K to determine the intra- or extracellular location of eGFP. The proteins were immunodetected using a GFP specific antibody. The integrity of the spheroplasts during the Proteinase K treatment was determined by immunodetection of GroEL (cytoplasmic^70,71^ or inner-membrane associated^72^), either simultaneously or sequentially. The GroEL band is indicated with an asterisk. Full, uncut gel images are provided in Fig. S12. Numbers on the right indicate the positions of migration of the molecular mass markers (kDa).

We addressed these two main points through extensive experimental mapping of transmembrane topology. We expressed truncated versions of MecR1, with eGFP fused at the C-terminal of each one of the loops defined between the predicted transmembrane helices, according to Model 1 (Fig. 1b). Our first study was the fluorescence emission in *E. coli* of these MecR1-eGFP fusion proteins, and also the susceptibility of these proteins to Proteinase K proteolysis in spheroplasts (Fig. 2). eGFP was used as a fluorescent probe of the location of the loops of membrane proteins that are translocated by the Sec system, as is the case for MecR1 (Fig. S3). eGFP is fluorescent when it folds in the cytoplasm but is not fluorescent in the periplasm after its unfolded transport^37–39^. The fluorescence emission of the fusion proteins was measured both in whole cells (Fig. S4) and in membrane protein preparations (Fig. 2a). Expression levels were corroborated by immunodetection (Fig. S5). In agreement with Model 1, the fusions F1 and F5 emitted fluorescence (Fig. 2a) and eGFP was protected from Proteinase K proteolysis (Fig. 2b). These results indicate that eGFP was fused to a cytoplasmic loop of MecR1. Fluorescence microscopy assays showed a homogeneous localization of the fluorescent versions F1 and F5 of MecR1-eGFP in the membrane of *E. coli* (Fig. S6). Although whole cells and membranes expressing F3 showed spectra characteristic of eGFP (Figs. 2a and S4), the intensity was significantly lower in membranes. We interpret this low intensity as the result of proteolysis, with the accumulation of eGFP in the soluble fraction (Fig. S5). Despite the lower fluorescence intensity of F3, protection of eGFP from Proteinase K proteolysis indicated cytoplasmic location. For both F3 and F5, ProtK treatment gave rise to smaller C-terminal fragments that contained eGFP. The mass of the fragments resulting from proteolysis of F3 and F5 (around 35 and 45 kDa, respectively) was consistent with proteolysis by ProtK at Thr106, in the extracellular loop that precedes the third TM helix (Fig. S7). The proteins F2C and F6 did not fluoresce and were susceptible to complete degradation by ProtK, indicating that the eGFP domain in these two proteins was located in the periplasm, in agreement with Model 1. The absence of fluorescence emission in F2N and F4C, together with their susceptibility to ProtK, indicated a periplasmic location. This result suggested that Model 1 was incorrect. For F2N, the results suggested that the second hydrophobic region (comprising residues 36–58) would not be structured as a reentrant helix but would instead be a standard transmembrane helix. The fluorescence emission of F4N presented a maximum at the wavelength characteristic of eGFP (with low intensity in membranes as was the case for F3; Figs. 2 and S4) and suggested cytoplasmic location. However, unlike the case for F3, F4N was susceptible to ProtK. The accumulation of the 45 kDa C-terminal fragment of F5 in the ProtK assay indicated that, in this longer version, the loops where eGFP was fused in F4N and F4C were indeed located in the cytoplasm, in agreement with Model 1. Hence, the inconsistencies between Model 1 and the assays with F4N and F4C were likely an artifact resulting from the use of truncated versions of MecR1, especially considering that this region is at the core of a folded (gluzincin) domain.

**Figure 3:**
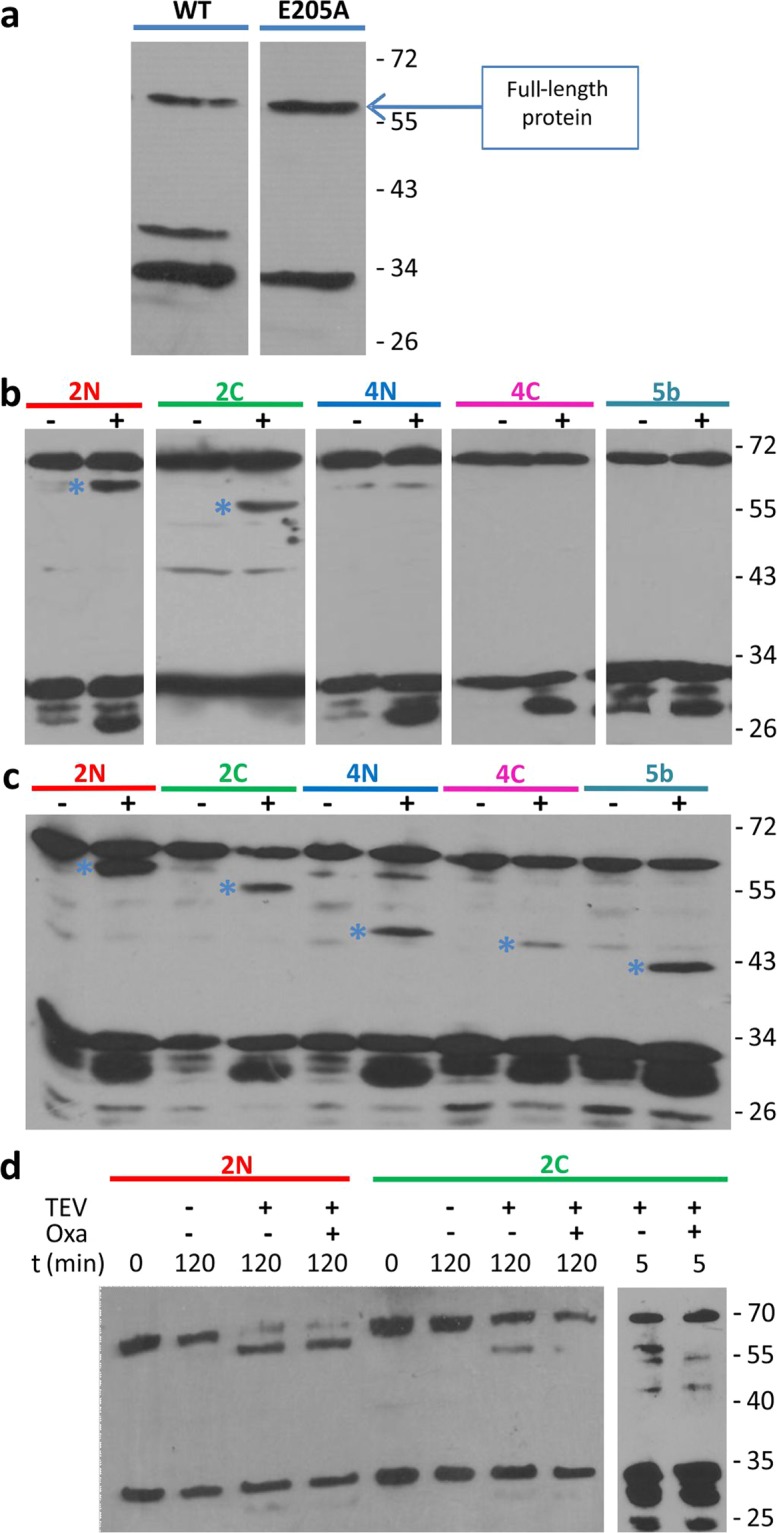
TEV Protease susceptibility assays of the MecR1.E205A.TEV insertional mutant proteins. (**a**) Expression of full-length MecR1 wild type and the E205A mutant (with a C-terminal His-tag) in *E. coli* BL21 Star (DE3), evaluated by Western blot using a His-tag specific HRP-conjugated antibody. Full, uncut gel images are provided in Fig. S13. (**b**) Spheroplasts expressing each MecR1.E205A.TEV insertional mutant protein were incubated in the absence (−) or presence (+) of TEV Protease, in order to determine the intra- or extracellular location of the TEV recognition peptide. (**c**) Membrane-protein extracts of *E. coli* expressing each MecR1.E205A.TEV insertional mutant protein were prepared and incubated in the absence (−) or presence (+) of TEV protease. In (**b**,**c**), the blue asterisk indicates the C-terminal fragment resulting from TEV proteolysis. The expected molecular weight of the full-length MecR1.E205A.TEV versions is 70.5 kDa. The expected molecular weight of the C-terminal His-tagged fragment after TEV treatment for each variant is: 2N, 61.3 kDa; 2C, 58.2 kDa; 4N, 47.3 kDa; 4C, 45.3 kDa and 5b, 40.1 kDa. (**d**) Spheroplasts expressing MecR1-TEV-2N and 2C were incubated in the presence (+) or absence (−) of oxacillin, prior to or simultaneously with TEV protease treatment. Lanes 2-4 and 6-8: incubation with oxacillin followed by incubation with 750 μg/ml TEV protease, 500 rpm. Lanes 9 and 10: simultaneous incubation with 100 μg/ml TEV protease, without agitation, as in *B*. Western blots were carried out using a His-tag specific HRP-conjugated antibody. Full, uncut gel images for *B* and *D* are provided in Figs. S14 and S15, respectively. Numbers on the right indicate the positions of migration of the molecular weight markers (kDa).

We then used full-length MecR1 to carry out a complementary topology assay using the highly sequence-specific cysteine protease from Tobacco Etch Virus (TEV Protease). Wild-type MecR1 (68.5 kDa, although it runs as 63–65 kDa) was partially proteolyzed in *E. coli*, and two C-terminal fragments of approximately 35 kDa and 30 kDa were observed. Mutation of the glutamic residue of the HEXXH metalloprotease motif to Ala (E205A mutation) resulted in the disappearance of the 35 kDa C-terminal fragment of MecR1 (Fig. 3a). Hence, the 35 kDa fragment could be due to auto-proteolysis (which would occur in the absence of antibiotics), as is the case for BlaR1^27,40^. This interpretation assigns a catalytic role to Glu205, as demonstrated for BlaR1. However, we cannot rule out the possibility that the WT and E205A variants differ in their susceptibility to a cellular protease. The 30 kDa fragment accumulated in the mutant protein and thus very likely arose from nonspecific proteolysis by an *E. coli* protease. Since the presence of this smaller fragment was not detrimental to our study, we used MecR1 E205A in the topology assay. We inserted the recognition site of the TEV Protease in the loops at the positions designated 2N, 2C, 4N, 4C and 5b of MecR1 (Fig. 1b), and we evaluated the susceptibility of these proteins to proteolysis in spheroplasts. We corroborated peptide 36–58 as a membrane-spanning segment, since both 2N and 2C were susceptible to TEV proteolysis as evidenced by the accumulation of 61 kDa and 58 kDa fragments, respectively (Fig. 3b). These results confirm the extracellular location of residues 69–102 (named loop L2 from now onwards). This information was used later to produce an improved structural model (Model 2, Fig. 1c). Constructs 4N, 4C, and 5b were not susceptible to TEV proteolysis in spheroplasts, consistent with a cytoplasmic location of these three loops (Fig. 3b). All three constructs were susceptible to TEV proteolysis in membrane preparations, confirming accessibility of the TEV sites in the cytoplasmic loops after cell lysis (Fig. 3c). Interestingly, when the MecR1.E205A.TEV.2C construct was pre-incubated with the β-lactam antibiotic oxacillin, there was no evident proteolysis by TEV protease (Fig. 3d). This was not the case for the other constructs tested. This change in TEV-susceptibility of the C-terminal of loop L2 (where the TEV recognition site is located in construct 2C) is consistent with conformational change triggered by the antibiotic.

**Figure 4:**
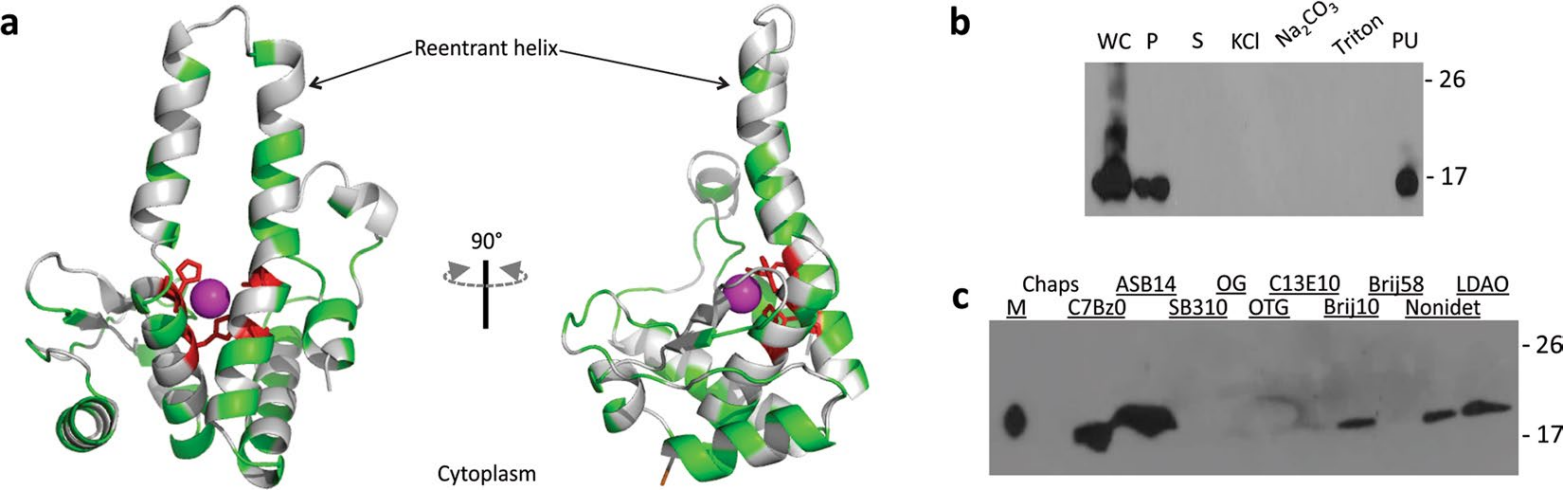
The gluzincin core of MecR1 is membrane-embedded. (**a**) The modeled gluzincin domain is shown, with hydrophobic residues displayed in light grey, polar residues in green and the putative metal ligands (H204, H208, E245 and D249) in red. (**b**) MecR1^GLZ^.E205A (19.8 kDa) was expressed in *E. coli* BL21 Star (DE3). IPTG-induced cells (WC, whole cell extract) were sonicated and centrifuged to separate the pellet (P; inclusion bodies and large fragments of membrane) from the fraction containing soluble proteins and membrane vesicles. The latter was subjected to ultracentrifugation to separate soluble proteins (S) from membrane proteins. Lanes labelled KCl, Na_2_CO_3_ and Triton correspond to fractions of proteins solubilized from the membrane preparation with 1 M KCl, 0.1 M Na_2_CO_3_ and 0.155% Triton X-100, respectively. Lane Pu is the pellet remaining after ultracentrifugation of the membranes treated with Triton X-100. (**c**) Detergent screening to evaluate conditions to solubilize MecR1^GLZ^.E205A from membranes. MecR1^GLZ^ was found in the membrane protein fraction (M). The fractions corresponding to solubilized proteins are shown. Both the soluble and insoluble fractions are shown in Fig. S8. MecR1^GLZ^.E205A could only be solubilized from membranes with the detergents C7Bz0, ASB-14, Brij10, Nonidet and LDAO, being the membrane solubilizing zwitterionic detergent ASB-14 (amidosulfobetaine-14) the one that allowed the highest level of protein recovery. In (**b**,**c**), MecR1^GLZ^.E205A was detected by Western blot using a His-tag specific HRP-conjugated antibody. Numbers on the right indicate the positions of migration of the molecular mass markers (kDa).

A key aspect of Model 1, especially evident when aligned to the Ste24p structure relaxed in a membrane, is that the transmembrane domain includes an amphipathic reentrant helix that is inserted in the gluzincin core between the HE_205_LSH and E_245_KVCD putative zinc-binding motifs (Fig. 4a). This reentrant helix (residues 213–243, which turns at I^226^FWFNP) corresponds roughly to the fifth hydrophobic segment predicted by TopPred and TMpred (Table 1). Consistent with this aspect of the model, the gluzincin region of the metalloprotease domain of MecR1 E205A (residues 147–304; denominated MecR1^GLZ^) interacted with the membrane through hydrophobic interactions (Fig. 4b). As expected from the model, we found that MecR1^GLZ^ was located in the membrane fraction (M, Fig. 4c). Treatment of membranes with KCl did not extract MecR1^GLZ^ from membranes^41^, thus indicating that it is not a loosely associated peripheral protein (Fig. 4b). The fraction of peripheral proteins associated by strong electrostatic interactions (as probed by Na_2_CO_3_ extraction) did not include MecR1^GLZ^ either, indicating that it might interact with lipids or proteins in the membrane through hydrophobic interactions. MecR1^GLZ^ was only solubilized from membranes with the detergents C7Bz0, ASB-14, Brij10, Nonidet, and LDAO (Figs. 4c and S8), although most of the protein remained insoluble. Addition of 10% V/V glycerol improved the amount of soluble MecR1^GLZ^ recovered from membranes with ASB-14, and MecR1^GLZ^ was purified as a folded domain in the presence of detergent (Fig. S9). These results show for the first time that the MecR1 gluzincin core is tailored to be membrane-associated. Therefore, in the initial stages of activation, before the accumulation of MecR2 allows for degradation of MecI by other proteases^23^, MecR1-dependent proteolysis of MecI would require that MecI diffuses to the membrane.

### A full-length MecR1 model revealing key interactions between the transmembrane and sensor domains

**Figure 5:**
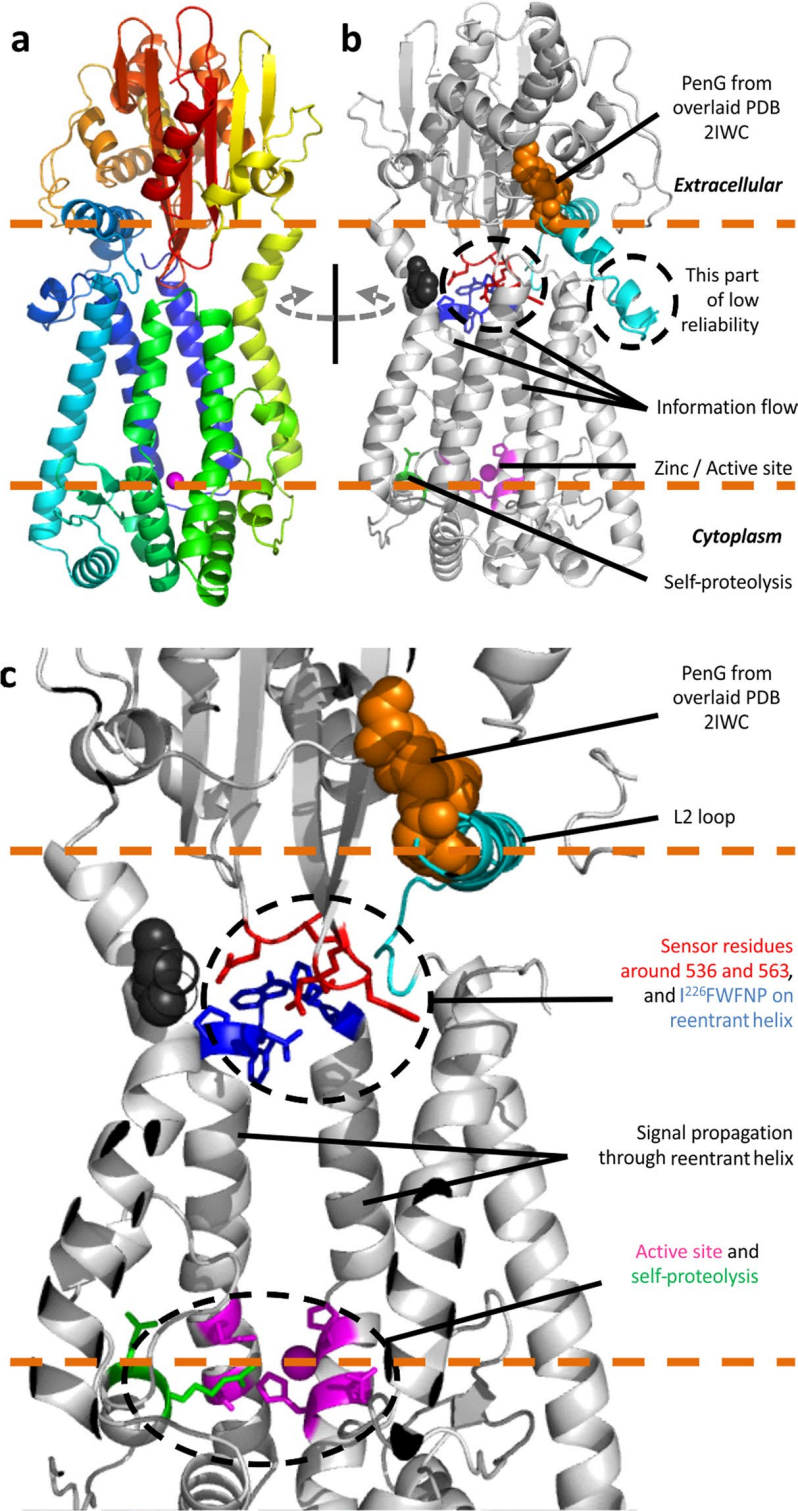
Model of full-length MecR1 (Model 2). (**a**) The full structure shown as cartoons colored from blue to red (N-terminal to C-terminal). (**b**) Structure rotated by 180° around the membrane normal, showing important elements, zoomed in with a cut-through view in (**c**). The dashed orange lines delineate membrane polar heads that guide protein insertion.

We corrected Model 1 of the transmembrane domain of MecR1to account for the following key results. Peptide 36-58 is a transmembrane helix instead of a reentrant helix, and loop L2 is extracellular and not a transmembrane helix. We also included in the modeling process the prediction that the L2 loop folds as a partially amphipathic helix upon binding to the sensor domain, a conclusion supported by NMR data for the homologous protein BlaR1^42^, and used this information and an X-ray structure of the sensor domain of MecR1 to build a model of the full-length protein. We dubbed this last model Model 2, shown in detail in Fig. 5.

More precisely, we first remodeled residues 1-340 of Model 1 by extending its reentrant helix as a straight transmembrane helix (36–58), spanning the full membrane (Fig. 1c). At this stage of the modeling pipeline we skipped the segment from residue 63 to 103, which includes part of the L2 loop modeled later on. We also stretched the transmembrane helix that connects the metalloprotease and sensor domains (potential helix 6 in Table 1, residues 315 to 338, which Robetta placed kinked by 90° at Pro331 leaving the sensor domain right inside the membrane). We shifted the position of this TM helix such that it could help to better close the hydrophilic chamber. The repositioning of this helix in this way leaves, upon connection of the MecR1 sensor domain, the β-lactam binding site right in front of the C-terminal end of TM helix 2 and the N-terminal end of TM helix 3, where the connecting L2 loop should locate.

Connection of the transmembrane and sensor domains relied on (i) the finding that the end of the last TM helix (TM6, 315–338) is observed in one of several X-ray structures of the sensor domain of MecR1 (PDB ID 2IWC^43^, starting at Ser334), and (ii) the NMR-based mapping of the interaction between the L2 loop of BlaR1 and its sensor domain^42^. This NMR study revealed further that L2 binds the sensor domain as an amphipathic alpha helix, through its polar side. The L2 loops of both BlaR1 and MecR1 are short, low complexity peptides, predicted highly disordered. Binding of L2 in a folded conformation to BlaR^S^, as found by the NMR study, resembles the situation found for molecular recognition features in other disordered yet functional regions of proteins^44^. Although the L2 loops of BlaR1 and MecR1 differ in sequence (Fig. S10), they both have amphipathic character. We built an idealized helix from the L2 sequence of MecR1, and docked its polar side to the sensor domain based on the residues of the sensor domain of BlaR1 identified by NMR, transferred from BlaR1 to MecR1. We next placed this L2-MecR^S^ complex on the revised model of the TM domain (TMD), guided by matching the N-terminal residues of the sensor domain and the last TM helix (TM6). In this operation, the L2 loop in a helical conformation results roughly centered between the C- and N-terminal ends of TM helices 2 and 3, suggesting that this location is approximately correct. This complex served as a base template for subsequent modeling, including all residues for which we have structural information, located in a way that satisfies sequence continuity and all the known transmembrane topology and interactions between sensor and effector domains. We finally used this TMD-L2-MecR^S^ complex as a template for homology-based modeling, leading to cleaner secondary structures especially with respect to interconnecting loops and turns, while removing strong clashes. Our final model (Model 2) is shown in Fig. 5. This refined model accounts for all of the structural information available from predictions, from existing experimental results, and from our experimental mapping of the topology of the transmembrane domain. The transmembrane domain of MecR1 displays a mostly hydrophobic surface that matches to the hydrophobic portion of the membrane lipids, with a rim of exposed positive residues on the cytoplasmic side and its three tryptophan residues (W40, W113, W228) near the polar lipid heads, as expected for transmembrane proteins^45,46^, and a hydrophilic chamber upholstered with polar residues. Interaction with the sensor domain closes the hydrophilic chamber on the extracellular leaflet of the membrane, leaving mostly polar and charged residues exposed to the extracellular space. The putative site of auto-proteolysis in MecR1, as inferred from results on the homologous protein BlaR1^19,27^, is close to the active site.

## Discussion

The membrane proteins BlaR1 and MecR1 have not been amenable to overexpression, purification and crystallization. As a result, our understanding of the molecular events that lead to their activation is limited. Our results provide insight to these molecular events. MecR1 has four full transmembrane helices (defined approximately by residues 5–28, 36–58, 110–135, and 315–338) and a reentrant helix (213–243). Both the N- and C-terminus of this helix are located in the cytoplasm, as the helix kinks and turns at the I^226^FWFNP loop. The four transmembrane helices and the reentrant helix form a transmembrane domain. The gluzincin core of the metalloprotease domain (historically known as the cytoplasmic domain) associates with the membrane through hydrophobic interactions. The reentrant helix (residues 213–243) is proposed to anchor this domain. This reentrant helix and the four transmembrane helices define an internal hydrophilic chamber wherein the zinc site sits at its base, as is seen also in the Ste24p metalloproteases^34^. Two histidine residues of the HE_205_LSH motif and the glutamic acid of the sequence motif E_245_KVCD would be ligands of the zinc atom, in agreement with results for BlaR1 from *B. licheniformis*^28^. The fourth ligand would be the hydrolytic water. Our mutagenesis results propose Glu205 as the catalytic base. As is seen in related metalloproteases, Glu205 polarizes the zinc-bound water molecule for nucleophilic attack on the scissile peptide bond, and then transfers a proton to the leaving group of the substrate^29,47,48^. Our proposed zinc ligands in MecR1 differ from those found in minigluzincins, where the Zn(II) ion is coordinated by the two histidines of the HEXXH motif and by a third histidine^49^.

In our model, the apo-sensor domain effectively seals the hydrophilic chamber, taking the role of the longer and more numerous TM helices of Ste24p-like metalloproteases. The sensor domain lies right above the reentrant helix at the core of the gluzincin domain. This interaction is reinforced through binding of the L2 helix of the metalloprotease domain to the sensor domain, and is in agreement with single-molecule force spectroscopy assays that indicate a strong interaction between the sensor domain and the metalloprotease domain of the homologous protein BlaR1 from *B. licheniformis*^50^. Two observations lead us to propose that the model shown in Fig. 5 corresponds to the apo-sensor protein (that is, the inactive state prior to interaction with the β-lactam). First, the site of acylation by the β-lactam antibiotic is hindered by the binding of the L2 helix to the sensor domain. Hence, the interaction of the antibiotic with the sensor domain requires dissociation of the helix L2 from the active site groove. Second, the metal site of the metalloprotease domain is too occluded to proteolyze its substrate, MecI, which suggests that our model corresponds to the inactive conformation. The transmembrane architecture of our structural model makes sense given our assumption that the protein is monomeric, as so far is observed for related metalloproteases. This assumption is supported by some of the top predictor models for MecR1, which was released as a mock target for modeling in the 13^th^ edition of the Critical Assessment of Structure Prediction (CASP) (Fig. S11)^51^. However, other CASP models suggest alternative arrangements of the transmembrane helices and gluzincin domain into a half-chamber with its polar residues exposed to the hydrophobic portion of the membrane. This arrangement would only make sense in the context of an oligomer that closes the chamber (Fig. S11).

Our model leads to a proposed mechanism for signal transduction. Acylation of the sensor domain must somehow perturb the metalloprotease domain in order to activate it. How might this proceed? On one hand, NMR studies on *S. aureus* BlaR1 suggest that acylation of Ser391 alters the dynamics of the sensor domain’s core β strands, around residues 536 and 563^52^. According to our model, these altered regions are within reach of the I^226^FWFNP turn in the reentrant helix of the gluzincin motif, between active site residues HE^205^LSH and E^245^KVCD. This interaction is also possible in some of the CASP13 models, consistent with oligomeric arrangements. The connection between the β-strands of the sensor domain and the I^226^FWFNP turn provides a clear route for transmission of perturbed dynamics, through the reentrant helix directly to the metal site. Based on the observation that serine acylation at the sensor domain of BlaR1 from *B. licheniformis* detaches it from the L2 loop^50,53^, and our results that indicate a reduction in the susceptibility to proteolysis of the C-terminal end of the L2 loop in the presence of β-lactam antibiotics, we propose possible routes for subsequent activation of proteolytic action. When the sensor domain detaches from L2 due to steric overlap with the acylated β-lactam (orange spheres in Fig. 5), it is free to hinge around the end of the last TM helix (possibly around Pro331, and/or the flexible residues of the N-terminal end of the sensor domain). Dislodgment of the sensor from the hydrophilic chamber will perturb its polar interior, and trigger the requisite conformational change. The nature of such conformational change is yet to be determined, but our model hints at a possible signal transduction mechanism. The transmembrane helices may adjust on the extracellular side so as to hide their internal polar residues from the hydrophobic membrane environment, with a concomitant widening and thus opening of the cavity on the cytoplasmic side. The amphipathic L2 helix may also rearrange, likely embedding partially in the membrane in this event as observed in some of the CASP13 models and consistent with higher resistance to proteolysis observed experimentally. Whatever the exact rearrangement, it must end up affecting the local structure around the metal site to enable substrate accessibility and catalysis. Such rearrangement could involve either direct exposure of the gluzincin core towards the cytoplasm, or opening of a crack in the transmembrane domain itself as suggested for ZMPSTE24^34^. In conclusion, this study provides the first coherent model of the structure of MecR1, opening routes for future functional investigations on how β-lactam binding culminates in the proteolytic degradation of MecI.

## Methods

### Strains, plasmids and reagents

All chemicals were of the highest quality available from Sigma-Aldrich, BioRad, Thermo Fisher Scientific, Abcam, and Promega. *Escherichia coli* DH5α (F^−^ φ80*lac*ZΔM15 Δ(*lac*ZYA-*arg*F)U169 *rec*A1 *end*A1 *hsd*R17(r_K_^−^, m_K_^+^) *pho*A *sup*E44 λ^−^ *thi*-1 *gyr*A96 *rel*A1) cells were used for transformation with ligation mixtures for cloning, and for transformation with plasmids for storage as frozen stocks. *E. coli* BL21 Star (DE3) cells (F^−^*omp*T *hsd*SB (rB^−^, mB^−^) *gal dcm rne*131 (DE3)) were employed for protein production from genes cloned in pET24a(+). *E. coli* MC4100 cells (*F*^−^*araD139 ∆(argF-lac) U169 rpsL150 relA1 deoC1 ptsF25 rpsR flbB301*)^54^ were employed for protein production from genes cloned in plasmid pMBLE^55^, under control of a lactose-inducible *tac* promoter. Two derivatives of strain *E. coli* MC4100, lacking the two main translocation systems, the *secA51* thermosensitive mutant (MC4100; *secA51*(Ts) *leuB*::Tn*10*)^56^ and *∆tatC* mutant (MC4100; *∆tatC*)^57^ were also used for protein production from genes cloned in plasmid pMBLE. Luria-Bertani (LB) broth (Difco) was used as growth medium for all bacterial strains.

### Genes and oligonucleotides

All synthetic genes, optimized for expression in *E. coli*, were purchased from Genscript. The *mecR1* gene from *S. aureus* N315 (NRS70) was synthesized with NdeI and BamHI restriction sites for cloning. The *egfp* gene was ordered with BamHI and HindIII sites for cloning. Oligonucleotides (Table S1) were purchased from either Invitrogen or Genbiotech.

### DNA techniques and cloning procedure

DNA preparation and related techniques were performed according to standard protocols^58^. Plasmid DNA was isolated using the Wizard Plus SV Minipreps Kit (Promega). Purification of PCR products and of plasmids from agarose gels was carried out using the Wizard SV Gel and PCR Clean-Up System Kit (Promega). All restriction enzymes were purchased from Promega. T4 DNA Ligase (Promega) and Platinum Pfx DNA Polymerase (Thermo Fisher Scientific) were used according to the user manuals. The sequence of the constructs was verified by the DNA Sequencing Facility at the University of Maine (https://umaine.edu/dnaseq/).

### Cloning and expression of the MecR1-eGFP versions

In order to achieve similar levels of expression in the membrane, the corresponding genes were cloned either in the expression vector pMBLE^55^ or in pET24a(+), and the proteins were expressed in *E. coli* MC4100 or BL21 Star (DE3), respectively. The *egfp* gene was cloned in vector pET24a(+) or in pMBLE between the BamHI and HindIII sites, giving rise to plasmids pET24a(+)::*egfp* and pMBLE::*egfp*, respectively. The DNA fragments coding for the different truncated versions of MecR1 were amplified by PCR using the *mecR1* synthetic gene as a template, using oLL18 as the forward primer and oLL19, oLL57, oLL20, oLL21, oLL46, oLL22, oLL23, or oLL24 as reverse primers for the *mecR1*-F1 (MecR1_1-S_36__eGFP), -F2N (MecR1_1-D_73__eGFP), -F2C (MecR1_1-T_102__eGFP), -F3 (MecR1_1-H_161__eGFP), -F4N (MecR1_1-K_196__eGFP), -F4C (MecR1_1-D_213__eGFP), -F5 (MecR1_1-I_268__EGFP) and -F6 (MecR1_1-Q_338__eGFP) constructs, respectively. Each PCR amplification product was isolated, digested with NdeI and BamHI, and finally purified from agarose gels. The fragment was ligated into either the pET24a (+)::*egfp* or pMBLE::*egfp* plasmid digested with NdeI and BamHI. CaCl_2_-chemically competent *E. coli* DH5α cells were transformed with each ligation mixture. Transformant colonies were selected in LB-kanamycin-agar plates. The constructs designated pMBLE::*mecR1-egfp* F1 to F6 allowed isopropyl β-D-1-thiogalactopyranoside (IPTG)-inducible expression of the MecR1 truncated versions fused to the N-terminal of eGFP in *E. coli* from the *tac* promoter. *E. coli* MC4100 cells harboring each pMBLE::*mecR1-egfp* vector were grown overnight in LB broth supplemented with 50 µg mL^−1^ kanamycin, at 37 °C and at 220 rpm. Fresh medium was inoculated with the overnight culture (dilution of 1/100) and grown at 37 °C until the OD_600_ reached 0.4, at which point the cultures were rapidly cooled to 28 °C and induced with 10 µM of IPTG. The cells were grown 4 h at 28 °C and at 220 rpm. The construct designated pET-24a(+)::*mecR1-egfp* F6 allowed IPTG-inducible expression of the MecR1 truncated version fused to the N-terminal of eGFP in *E. coli* from the T7 promoter. *E. coli* BL21 Star (DE3) cells harboring each the corresponding vector were grown overnight in LB broth supplemented with 50 µg mL^−1^ kanamycin, at 37 °C and at 220 rpm. Fresh medium was inoculated with the overnight culture (dilution of 1/100) and grown at 37 °C until the OD_600_ reached 0.8, at which point the cultures were rapidly cooled to 20 °C and induced with 500 µM of IPTG. The cells were grown overnight (16–20 h) at 20 °C and at 220 rpm.

### Cloning and expression of MecR1.E205A and of the MecR1.E205A-TEV insertional mutants

The gene coding for MecR1.E205A was engineered using the overlap extension PCR protocol^59^. The 5′-end of the gene was PCR amplified with the forward primer oLL18 and the reverse mutagenic primer oLL54. The 3′-end of the *mecR1* gene was PCR amplified with the reverse primer oLL26 and the forward mutagenic primer oLL53. Mutagenic primers were designed in order to hybridize to each other. Both PCR reactions were purified using Wizard SV Gel and PCR Clean-Up System kit (Promega) and quantified (Nanodrop 2000, Thermo Fisher Scientific). In a third PCR reaction, equal amounts of both products (40 ng) were used as templates and amplified using external primers oLL18 and oLL26, which introduce an NdeI and an XhoI restriction site, respectively. Primer oLL26 was designed without the *mecR1* stop codon, in order to clone the gene in pET24a(+) in phase for the expression of MecR1 E205A with a C-terminal His-tag. The PCR amplification product was purified and digested with NdeI and XhoI restriction enzymes, and purified from agarose gels. The DNA fragment was then ligated into pET24a(+) digested with the same restriction enzymes. Transformation with the ligation mixture and selection was done as described above.

A sequence coding for the 7 amino acids corresponding to the TEV protease recognition site (ENLYFQG), followed by two flexible amino acids, (GS) was inserted right after the codons coding for amino acids S72 (2N), E101 (2C), D195 (4N), H212 (4C) and I255 (5b) of MecR1 E205A. Insertional mutants 2N, 2C, 4N and 4C were obtained using the overlap extension PCR protocol. External primers used were oLL18 (forward) and oLL26 (reverse). Internal mutagenic primers (reverse primer in the reaction to amplify the 5′-end of the *mecR1.E205A* gene and forward primer in the reaction to amplify the 3′-end of the gene) were as follows: oLL67 and oLL68 for 2N; oLL69 and oLL70 for 2C; oLL71 and oLL72 for 4N; oLL73 and oLL74 for 4 C. The 5′end of each mutagenic primer codes for the TEV recognition site. The final PCR amplification product was cloned in pET24a(+), between the NdeI and XhoI sites, as described for *mecR1.E205A*. A modified protocol was used to generate the gene coding for the 5b mutant: the 5′-end of the mutagenic primer codes for the TEV recognition site amino acid sequence and the codons coding for the two flexible amino acids (GS) introduce a BamHI restriction site, which was then used for reconstitution of the gene. One PCR reactions was carried out to amplify the 5′-end of the *mecR1*.*E205A* gene using primers oLL18 (forward) and the mutagenic reverse primer oLL90; the amplification product was purified, digested with NdeI and BamHI and cloned in pET24a(+) digested with the same enzymes. A second PCR reaction was carried out to amplify the 3′-end of *mecR1*.*E205A* using the mutagenic forward primer oLL91 and the reverse primer oLL26. The purified PCR fragment was digested with BamHI and XhoI and cloned in pET24a(+), downstream of the 5′-end of *mecR1.E205*, between the BamHI and XhoI sites. Transformation with the ligation mixture and selection was done as previously described.

MecR1.E205A and the MecR1.E205A-TEV insertional mutants (with a C-terminal His-tag, LEHHHHHH) were expressed in *E. coli* BL21 Star (DE3) cells harboring the corresponding pET-24a(+) construct. The transformant cells were grown overnight in LB broth supplemented with 50 µg mL^−1^ kanamycin, at 37 °C and at 220 rpm. Fresh medium was inoculated with the overnight culture (dilution of 1/100) and grown at 37 °C until the OD_600_ reached 0.7, at which point the cultures were rapidly cooled to 20 °C and expression of the recombinant proteins was induced with 5 µM IPTG at 20 °C for 16 h, 220 rpm.

### Cloning and expression of MecR1^GLZ^

For this construction, the coding DNA fragment was PCR-amplified using the *mecR1.E205A* gene as the template and primers oLL40 and oLL84, which introduce an NdeI and an XhoI restriction site, respectively. Primer oLL84 was designed without including a stop codon, in order to clone the gene in pET-24a(+) in phase for the expression of with a C-terminal His-tag. The PCR amplification product was purified, digested with NdeI and XhoI, and purified from agarose gels. The DNA fragment was then ligated into pET-24a(+) digested with the same restriction enzymes. Transformation with the ligation mixture and selection was done as previously described. Expression was carried out as described for MecR1.E205A.

### Spheroplasts preparation

All steps were performed either in water-ice bath or at 4 °C. The induced culture expressing the protein of interest was cooled down and washed once with Wash Buffer A (20 mM Tris-HCl, pH 8.0, 150 mM NaCl). Next, the cells were pelleted by centrifugation for 6 min at 2,700 *g*, 4 °C and resuspended in a volume of Lysozyme Buffer (20 mM Tris-HCl, pH = 8.0, 0.1 mM ethylenediaminetetraacetic acid (EDTA), 20% w/v sucrose, 1 mg mL^−1^ Lysozyme, 0.5 mM phenylmethylsulfonyl fluoride (PMSF)) that was calculated as follow: ***V*** = (OD_600_ * initial culture volume)/10. The cells were incubated in this buffer for 30 min at 4 °C with gentle rocking at 12 rpm. After that, the spheroplasts were pelleted by centrifugation for 4 min at 2,700 *g* and at 4 °C, and resuspended in a volume ***V*** of Wash Buffer B (20 mM Tris-HCl pH 8.0 buffer, 20% w/v sucrose), pelleted by centrifugation and resuspended in a volume ***V*** of Proteolysis Buffer (20 mM Tris-HCl pH 8.0 buffer, 10 mM CaCl_2_).

### Membrane protein preparations

The cells in 50 mL of the corresponding induced culture were cooled in ice and harvested by centrifugation for 5 min at 5,000 *g*, 4 °C. Cells were washed with Buffer A (100 mM sodium phosphate, 50 mM sodium bicarbonate, pH 7.5) and resuspended in 5 mL of Buffer A. After addition of 5 µM PMSF, the cell contents were liberated by sonification in an ice-water bath (GEX 600 Ultrasonic Processor, Cole-Parmer: 5 cycles of 30 s sonication at 20% amplitude each, with a 90 s break in between). The extracts were centrifuged for 20 min at 5,000 *g* and at 4 °C. The pellet from ultracentrifugation of the supernatant (1 h at 150,000 *g* at 4 °C) gave the membrane fraction. This fraction was resuspended in 500 µL of Buffer A.

### Proteinase K susceptibility assays

The genes coding for fusions F1-F5 were cloned in pMBLE and the proteins were expressed in *E. coli* MC4100. Due to the lower level of expression of fusion F6, this gene was cloned in pET24a(+) and the protein was expressed in *E. coli* BL21 Star (DE3). Spheroplasts were suspended in Proteolysis Buffer and incubated on ice for 30 min without agitation, in the presence (or absence) of 100 µg mL^−1^ Proteinase K (Sigma). The reaction was stopped by addition of PMSF (5 mM final concentration) and centrifugation (3 min at 3,200 *g*). The pellet was resuspended in the same volume of Protein Loading Buffer (12 mM Tris-HCl pH 6.8 buffer, 5% v/v glycerol, 0.4% SDS, 2.88 mM 2-mercaptoethanol, 0.02% w/v bromophenol blue, 5 mM PMSF), and boiled immediately for 5 minutes. Each lane in the SDS-PAGE gel was loaded with spheroplasts corresponding to 2 × 10^8^ cells.

### TEV protease susceptibility assays

Tobbaco etch virus (TEV) protease was purified as described by Phan *et al*.^60^. Spheroplasts prepared from *E. coli* BL21 Star (DE3) cells harboring the corresponding expression vector were suspended in Proteolysis Buffer and were incubated with 100 µg mL^−1^ TEV protease for 5 min at 34 °C without agitation. Membrane-protein extracts, from *E. coli* cells expressing each MecR1.E205A.TEV insertional mutant protein, were prepared and incubated without (−) or with (+) 500 µg mL^−1^ of TEV protease for 2 h at 34 °C, and at 1000 rpm. Conformational changes triggered by oxacillin were initially evaluated by simultaneous incubation of the antibiotic and the protease. Spheroplasts were incubated for 5 min at 34 °C without agitation with 100 µg mL^−1^ TEV protease, in the absence or presence of 455 μM oxacillin. In addition, the protease assay was repeated after preincubation with oxacillin. Spheroplasts were prepared and incubated for 15 min at 37 °C, in the presence (or absence) of 455 μM oxacillin. Afterwards the spheroplasts were incubated for 2 h at 34 °C and at 500 rpm, in the presence (or absence) of 750 μg mL^−1^ of TEV protease. The latter conditions of the protease assay were harsher, in order to assure proteolysis if the TEV recognition site was accessible. In the case of analysis of the proteins in spheroplasts, total protein extracts corresponding to 2 × 10^8^ cells were loaded. In all cases, the reaction was stopped by addition of concentrated Protein Loading Buffer and boiled immediately for 5 min.

### Fluorescence spectroscopy

The fluorescence emission spectra of the MecR1-eGFP fusions were recorded with excitation at λ_exc_ = 490 nm and λ_em_ 500–600 nm (Cary Eclipse Fluorescence Spectrophotometer, Agilent). For assays carried out with whole cells suspensions, *E. coli* cells expressing each one of the different MecR1-eGFP fusions, or transformed with the empty vector, were washed twice with Phosphate buffer (10 mM NaH_2_PO_4_ pH 7.4 buffer, 280 mM NaCl, 6 mM KCl). Cells were suspended in an appropriate volume of PBS buffer to give an OD_600_ = 1. Membrane protein preparations were carried out as described above for assays carried out with suspensions of membrane vesicles. The amber membrane pellet obtained after ultracentrifugation was resuspended in 500 µL of Phosphate buffer and its protein concentration was quantified using the Pierce BCA Protein Assay Kit (Thermo Fisher Scientific). Samples were diluted in PBS to a final concentration of 40 µg mL^−1^ of total protein for measurement.

### Solubilization of MecR1^GLZ^ from *E. coli* membrane preparations

We used a described protocol to sequentially extract membrane proteins^41^. Total membranes were pelleted by ultracentrifugation at 4 °C for 1 h at 150,000 *g*. Membranes were resuspended in 1 M KCl, incubated for 30 min on ice, and ultracentrifuged to obtain loosely associated peripheral proteins in the supernatant. The peripheral proteins associated by strong electrostatic interactions were released by treatment of the remaining membranes with 0.1 M Na_2_CO_3_ for 30 min on ice followed by ultracentrifugation, to pellet the remaining membranes with the integral or hydrophobically associated proteins. This final pellet was treated with Triton X-100 (0.155% w/v), to extract integral or hydrophobically associated proteins in detergent micelles. Different detergents were evaluated for their capacity to solubilize MecR1^GLZ^ from membranes. Membranes were prepared as described above. After ultracentrifugation for 1 h at 150,000 *g* and 4 °C, the membranes were suspended using a Potter-Elvehjem PTFE (Sigma-Aldrich) in 1 mL of 10 mM NaH_2_PO_4_, 600 mM NaCl, 6 mM KCl, 0.1 µg/ml Pefabloc SC (Sigma-Aldrich), pH 7.4 buffer. Aliquots (100 µL) were supplemented with detergent (800 µL, from Sigma-Aldrich) to reach a final concentration (w/v) of: 3.7% Chaps, 2% C7Bz0, 2% ASB-14, 0.01% SB3_10_, 7.3% OG, 0.5% OTG, 0.5% C_13_E_10_, 0.5% Brij-10, 0.5% Brij-58, 0.5% Nonidet P40 or 0.5% LDAO. The resulting suspensions were incubated overnight at 4 °C with gentle rocking (12 rpm). Subsequently, the samples were centrifuged for 30 min at 21,000 *g* and at 4 °C, to separate the solubilized protein fraction from the pellet of insoluble-precipitated proteins. The effectiveness of each detergent to solubilize the protein was evaluated by SDS-PAGE gel analysis using Western blots (as described below) for detection of protein.

### Western blot assays

The protein samples were resolved in 12% Tris-glycine SDS-PAGE. After electrophoresis, the proteins were transferred to 0.45 µM nitrocellulose membranes (BioRad) using Transference Buffer (25 mM Tris, 192 mM Glycine, 20% Methanol, pH 8.3 buffer) in a Trans-Blot system (BioRad). The membranes were blocked overnight at 4 °C by incubation with 10 mL of Blotto (100 mM Tris-HCl pH 7.5 buffer, 150 mM NaCl, 3% bovine serum albumin, 3% non-fat milk, 0.02% sodium azide). The eGFP fusion proteins were detected by immunoblot analysis using a rabbit GFP-specific primary antibody (ab290, Abcam; 1:10,000 dilution) and a 1:7,000 dilution of the goat anti-rabbit HRP-conjugated secondary antibody (#32460, Thermo Fisher Scientific). For detection of the control protein GroEL, the membranes were either co-incubated or separately incubated with a rabbit GroEL specific primary antibody^61^ (1:80,000 dilution). The C-terminal His-tag of wild-type MecR1, MecR1.E205A, the MecR1.E205A TEV-insertional mutants, and MecR1^GLZ^ were detected by Western bot using a rabbit His-Tag specific antibody conjugated to HRP (ab1187, Abcam; 1: 50,000 dilution). In all cases, the antibodies were diluted in Tween/Tris-buffered saline (T-TBS: 100 mM Tris-HCl pH 7.5 buffer, 150 mM NaCl, 0.1% Tween 20) with 1.2% nonfat dry milk. PageRuler Prestained Protein Ladder (Thermo Fisher Scientific) provided the molecular markers. The membranes were washed with T-TBS buffer. The enhanced chemiluminiscence system Super-Signal West Dura Extended Duration Substrate (Pierce) was used according to the manufacturer’s instructions to detect the immune complexes, together with CL-XPosure films (Thermo Fisher Scientific).

### Molecular modeling and simulations

“Model 1” was the top model delivered by the Robetta server (standard settings as of April 2017)^62^ on MecR1’s full sequence. Robetta split the protein into 2 large domains connected through the last TM helix. In the main text we discuss the TM domain from this model as the focus of our attention. EVFold^36^ (standard settings as of April 2017) computed the evolutionary couplings of the 1–340 segment of MecR1. We consider subsequently a refined MecR1 model, including a TM domain corrected based on our experimental map of the TM topology. The operations for building “Model 2” described in the text were achieved through cycles of manual structure rebuilding in PyMOL, relaxed through atomistic simulations with the amber99sbILDN force field in implicit solvent. The resulting basic scaffold was then used as the template for homology modeling with the I-TASSER server^63^. The coordinates of our Model 2 and all CASP13-contributed models are available for download and online 3D visualization at http://lucianoabriata.altervista.org/modelshome.html. Membrane-protein systems for molecular dynamics simulations were built with the CHARMM-GUI server as of November 2014^64^. The coarse-grained simulation to stabilize ZMPSTE24’s structure in a POPE membrane was run with the MARTINI forcefield^65^ in Gromacs^66^. Atomistic simulations with CHARMM36^67^ used parameters for proteins and lipids, and TIP3P^68^ water in NAMD^69^. The protocols provided by the CHARMM-GUI server were used for equilibration and production simulations.

## Supplementary information

Supplementary Information

## Data Availability

The final structural model of MecR1 generated during the current study, and the MecR1 models obtained from CASP13 structure predictor groups, are available for direct download and online visualization at http://lucianoabriata.altervista.org/modelshome.html.

## References

[1] Boucher HW (2010). Challenges in anti-infective development in the era of bad bugs, no drugs: a regulatory perspective using the example of bloodstream infection as an indication. Clin Infect Dis.

[2] Daum RS (1998). Community-acquired methicillin-resistant staphylococcus aureus infections. Pediatr Infect Dis J.

[3] Loffler CA, Macdougall C (2007). Update on prevalence and treatment of methicillin-resistant Staphylococcus aureus infections. Expert Rev Anti Infect Ther.

[4] Chambers HF (2009). Pathogenesis of staphylococcal infection: a manner of expression. J Infect Dis.

[5] McKenna M (2012). Vaccine development: Man vs MRSA. Nature.

[6] Garza-Gonzalez E, Dowzicky MJ (2013). Changes in Staphylococcus aureus susceptibility across Latin America between 2004 and 2010. Braz J Infect Dis.

[7] Turner NA (2019). Methicillin-resistant Staphylococcus aureus: an overview of basic and clinical research. Nature reviews. Microbiology.

[8] Poulakou G, Lagou S, Tsiodras S (2019). What’s new in the epidemiology of skin and soft tissue infections in 2018?. Current opinion in infectious diseases.

[9] Livermore DM (2009). Has the era of untreatable infections arrived?. J Antimicrob Chemother.

[10] Gilbert DN (2010). The ‘10 × 20 Initiative’: Pursuing a Global Commitment to Develop 10 New Antibacterial Drugs by 2020. Clin. Infect. Dis..

[11] David MZ, Dryden M, Gottlieb T, Tattevin P, Gould IM (2017). Recently approved antibacterials for methicillin-resistant Staphylococcus aureus (MRSA) and other Gram-positive pathogens: the shock of the new. Int J Antimicrob Agents.

[12] Gajdács Márió (2019). The Continuing Threat of Methicillin-Resistant Staphylococcus aureus. Antibiotics.

[13] Talbot George H, Jezek Amanda, Murray Barbara E, Jones Ronald N, Ebright Richard H, Nau Gerard J, Rodvold Keith A, Newland Jason G, Boucher Helen W (2019). The Infectious Diseases Society of America’s 10 × ’20 Initiative (10 New Systemic Antibacterial Agents US Food and Drug Administration Approved by 2020): Is 20 × ’20 a Possibility?. Clinical Infectious Diseases.

[14] Banerjee R, Gretes M, Basuino L, Strynadka N, Chambers HF (2008). *In vitro* selection and characterization of ceftobiprole-resistant methicillin-resistant Staphylococcus aureus. Antimicrob Agents Chemother.

[15] Kelley WL, Jousselin A, Barras C, Lelong E, Renzoni A (2015). Missense mutations in PBP2A Affecting ceftaroline susceptibility detected in epidemic hospital-acquired methicillin-resistant Staphylococcus aureus clonotypes ST228 and ST247 in Western Switzerland archived since 1998. Antimicrob Agents Chemother.

[16] Chan LC (2015). Ceftobiprole- and ceftaroline-resistant methicillin-resistant Staphylococcus aureus. Antimicrob Agents Chemother.

[17] Llarrull LI, Fisher JF, Mobashery S (2009). Molecular basis and phenotype of methicillin resistance in Staphylococcus aureus and insights into new beta-lactams that meet the challenge. Antimicrob Agents Chemother.

[18] Peacock SJ, Paterson GK (2015). Mechanisms of Methicillin Resistance in Staphylococcus aureus. Annual review of biochemistry.

[19] Llarrull LI, Toth M, Champion MM, Mobashery S (2011). Activation of BlaR1 protein of methicillin-resistant Staphylococcus aureus, its proteolytic processing, and recovery from induction of resistance. J Biol Chem.

[20] Fuda CC, Fisher JF, Mobashery S (2005). Beta-lactam resistance in Staphylococcus aureus: the adaptive resistance of a plastic genome. Cell Mol Life Sci.

[21] Berger-Bachi B (1999). Genetic basis of methicillin resistance in Staphylococcus aureus. Cell Mol Life Sci.

[22] Clarke SR, Dyke KG (2001). The signal transducer (BlaRI) and the repressor (BlaI) of the Staphylococcus aureus beta-lactamase operon are inducible. Microbiology.

[23] Arede P, Milheirico C, de Lencastre H, Oliveira DC (2012). The anti-repressor MecR2 promotes the proteolysis of the mecA repressor and enables optimal expression of beta-lactam resistance in MRSA. PLoS Pathog.

[24] Golemi-Kotra D, Cha JY, Meroueh SO, Vakulenko SB, Mobashery S (2003). Resistance to beta-lactam antibiotics and its mediation by the sensor domain of the transmembrane BlaR signaling pathway in Staphylococcus aureus. J Biol Chem.

[25] Cha J, Vakulenko SB, Mobashery S (2007). Characterization of the beta-lactam antibiotic sensor domain of the MecR1 signal sensor/transducer protein from methicillin-resistant Staphylococcus aureus. Biochemistry.

[26] Rawlings ND, Barrett AJ (1995). Evolutionary families of metallopeptidases. Methods in enzymology.

[27] Zhang HZ, Hackbarth CJ, Chansky KM, Chambers HF (2001). A proteolytic transmembrane signaling pathway and resistance to beta-lactams in staphylococci. Science.

[28] Berzigotti S, Benlafya K, Sepulchre J, Amoroso A, Joris B (2012). Bacillus licheniformis BlaR1 L3 loop is a zinc metalloprotease activated by self-proteolysis. PLoS One.

[29] Hooper, N. M. Families of zinc metalloproteases. *FEBS Lett*, **354**, 1–6, doi:0014-5793(94)01079-X (1994). 10.1016/0014-5793(94)01079-x 7957888

[30] Thumanu K (2006). Discrete steps in sensing of beta-lactam antibiotics by the BlaR1 protein of the methicillin-resistant Staphylococcus aureus bacterium. Proc. Natl. Acad. Sci. USA.

[31] Wilke MS, Hills TL, Zhang HZ, Chambers HF, Strynadka NC (2004). Crystal structures of the Apo and penicillin-acylated forms of the BlaR1 beta-lactam sensor of Staphylococcus aureus. J. Biol. Chem..

[32] Borbulevych O (2011). Lysine Nzeta-decarboxylation switch and activation of the beta-lactam sensor domain of BlaR1 protein of methicillin-resistant Staphylococcus aureus. J. Biol. Chem..

[33] Llarrull LI, Prorok M, Mobashery S (2010). Binding of the gene repressor BlaI to the bla operon in methicillin-resistant Staphylococcus aureus. Biochemistry.

[34] Quigley A (2013). The structural basis of ZMPSTE24-dependent laminopathies. Science.

[35] Pryor EE (2013). Structure of the integral membrane protein CAAX protease Ste24p. Science.

[36] Marks DS, Hopf TA, Sander C (2012). Protein structure prediction from sequence variation. Nat Biotechnol.

[37] Feilmeier BJ, Iseminger G, Schroeder D, Webber H, Phillips GJ (2000). Green fluorescent protein functions as a reporter for protein localization in *Escherichia coli*. J Bacteriol.

[38] Drew D (2002). Rapid topology mapping of *Escherichia coli* inner-membrane proteins by prediction and PhoA/GFP fusion analysis. Proc Natl Acad Sci USA.

[39] McKenzie NL, Nodwell JR (2009). Transmembrane topology of the AbsA1 sensor kinase of Streptomyces coelicolor. Microbiology.

[40] Llarrull LI, Mobashery S (2012). Dissection of events in the resistance to beta-lactam antibiotics mediated by the protein BlaR1 from Staphylococcus aureus. Biochemistry.

[41] Chen X, Brown T, Tai PC (1998). Identification and characterization of protease-resistant SecA fragments: secA has two membrane-integral forms. J Bacteriol.

[42] Frederick TE, Wilson BD, Cha J, Mobashery S, Peng JW (2014). Revealing cell-surface intramolecular interactions in the BlaR1 protein of methicillin-resistant Staphylococcus aureus by NMR spectroscopy. Biochemistry.

[43] Marrero A, Mallorqui-Fernandez G, Guevara T, Garcia-Castellanos R, Gomis-Ruth FX (2006). Unbound and acylated structures of the MecR1 extracellular antibiotic-sensor domain provide insights into the signal-transduction system that triggers methicillin resistance. J Mol Biol.

[44] Mohan A (2006). Analysis of molecular recognition features (MoRFs). J Mol Biol.

[45] Ulmschneider MB, Sansom MS (2001). Amino acid distributions in integral membrane protein structures. Biochim Biophys Acta.

[46] Pilpel Y, Ben-Tal N, Lancet D (1999). kPROT: a knowledge-based scale for the propensity of residue orientation in transmembrane segments. Application to membrane protein structure prediction. J Mol Biol.

[47] Gao X (2010). Structural basis for the autoprocessing of zinc metalloproteases in the thermolysin family. Proc Natl Acad Sci USA.

[48] Le Moual H, Devault A, Roques BP, Crine P, Boileau G (1991). Identification of glutamic acid 646 as a zinc-coordinating residue in endopeptidase-24.11. J Biol Chem.

[49] Lopez-Pelegrin M (2013). A novel family of soluble minimal scaffolds provides structural insight into the catalytic domains of integral membrane metallopeptidases. J Biol Chem.

[50] Mescola A, Dauvin M, Amoroso A, Duwez AS, Joris B (2019). Single-molecule force spectroscopy to decipher the early signalling step in membrane-bound penicillin receptors embedded into a lipid bilayer. Nanoscale.

[51] Abriata LA, Tamo GE, Dal Peraro M (2019). A further leap of improvement in tertiary structure prediction in CASP13 prompts new routes for future assessments. Proteins.

[52] Staude MW (2015). Investigation of signal transduction routes within the sensor/transducer protein BlaR1 of Staphylococcus aureus. Biochemistry.

[53] Hanique S (2004). Evidence of an intramolecular interaction between the two domains of the BlaR1 penicillin receptor during the signal transduction. J Biol Chem.

[54] Checa SK, Viale AM (1997). The 70-kDa heat-shock protein/DnaK chaperone system is required for the productive folding of ribulose-biphosphate carboxylase subunits in *Escherichia coli*. Eur J Biochem.

[55] Gonzalez LJ (2016). Membrane anchoring stabilizes and favors secretion of New Delhi metallo-beta-lactamase. Nat Chem Biol.

[56] Bowers CW, Lau F, Silhavy TJ (2003). Secretion of LamB-LacZ by the signal recognition particle pathway of *Escherichia coli*. J Bacteriol.

[57] Bogsch EG (1998). An essential component of a novel bacterial protein export system with homologues in plastids and mitochondria. J Biol Chem.

[58] Sambrook, J., Fritsch, E. F. & Maniatis, T. *Molecular Cloning: A Laboratory Manual*. 2nd edn, (1989).

[59] Bryksin A, Matsumura I (2013). Overlap extension PCR cloning. Methods Mol Biol.

[60] Phan J (2002). Structural basis for the substrate specificity of tobacco etch virus protease. J Biol Chem.

[61] Moran-Barrio J, Limansky AS, Viale AM (2009). Secretion of GOB metallo-beta-lactamase in *Escherichia coli* depends strictly on the cooperation between the cytoplasmic DnaK chaperone system and the Sec machinery: completion of folding and Zn(II) ion acquisition occur in the bacterial periplasm. Antimicrob Agents Chemother.

[62] Kim DE, Chivian D, Baker D (2004). Protein structure prediction and analysis using the Robetta server. Nucleic Acids Res.

[63] Zhang Y (2008). I-TASSER server for protein 3D structure prediction. BMC Bioinformatics.

[64] Jo S, Kim T, Iyer VG, Im W (2008). CHARMM-GUI: a web-based graphical user interface for CHARMM. J Comput Chem.

[65] Marrink SJ, Risselada HJ, Yefimov S, Tieleman DP, de Vries AH (2007). The MARTINI force field: coarse grained model for biomolecular simulations. J Phys Chem B.

[66] Abraham MJ (2015). GROMACS: High performance molecular simulations through multi-level parallelism from laptops to supercomputers. SoftwareX.

[67] Best RB (2012). Optimization of the additive CHARMM all-atom protein force field targeting improved sampling of the backbone phi, psi and side-chain chi(1) and chi(2) dihedral angles. J Chem Theory Comput.

[68] Jorgensen WL, Chandrasekhar J, Madura JD, Impey RW, Klein ML (1983). Comparison of simple potential functions for simulating liquid water. The Journal of Chemical Physics.

[69] Phillips JC (2005). Scalable molecular dynamics with NAMD. J Comput Chem.

[70] DeLisa MP, Tullman D, Georgiou G (2003). Folding quality control in the export of proteins by the bacterial twin-arginine translocation pathway. Proc Natl Acad Sci USA.

[71] Marrichi M, Camacho L, Russell DG, DeLisa MP (2008). Genetic toggling of alkaline phosphatase folding reveals signal peptides for all major modes of transport across the inner membrane of bacteria. J Biol Chem.

[72] Torok Z (1997). Evidence for a lipochaperonin: association of active protein-folding GroESL oligomers with lipids can stabilize membranes under heat shock conditions. Proc Natl Acad Sci USA.

