# Supplementary material for: An experiment-informed signal transduction model for the role of the Staphylococcus aureus MecR1 protein in β-lactam resistance
Source: Sci Rep. 2019 Dec 20;9:19558. doi: 10.1038/s41598-019-55923-z (PMC6925264; doi:10.1038/s41598-019-55923-z)
Supplement: Supplementary file 1 — Supplementary Information [file 41598_2019_55923_MOESM1_ESM.pdf]

Supplementary Information

An experiment-informed signal transduction model for the role of the *Staphylococcus aureus* MecR1 protein in β-lactam resistance

Bruno S. Belluzo[1ξ], Luciano A. Abriata[2ξ], Estefanía Giannini[1ξ], Damila Mihovilcevic[1], Matteo Dal Peraro[2] and Leticia I. Llarrull[1,3*]

[1]Instituto de Biología Molecular y Celular de Rosario (IBR, CONICET-UNR), Predio CONICET Rosario, 27 de Febrero 210 bis, 2000 Rosario, Argentina; [2]Laboratory for Biomolecular Modeling - École Polytechnique Fédérale de Lausanne and Swiss Institute of Bioinformatics, CH-1015 Lausanne, Switzerland; [3]Área Biofísica, Facultad de Ciencias Bioquímicas y Farmacéuticas, Universidad Nacional de Rosario, Suipacha 570, 2000 Rosario, Argentina

[ξ]Equally contributing authors
[*]To whom correspondence should be addressed; E-mail: llarrull@ibr-conicet.gov.ar

This PDF file includes:

- Supplementary Text: Supplemental Materials and Methods;
- Figs. S1 to S16, where Figs. S12-S16 are the full, uncut gel images for Figures 2b, 3a, 3b, 3d and S3;
- Table S1;
- References for Supplemental Material citations.

SUPPLEMENTARY TEXT

Supplemental Materials and Methods

Fluorescence microscopy. In order to corroborate the localization of the MecR1-eGFP fusions in *E. coli* cells, the cells in 1 ml of the induced culture were pelleted by centrifugation for 3 min at 3,200 g, 4 °C, and resuspended in 1 ml of PBS (137 mM NaCl, 2.7 mM KCl, 8 mM Na_2HPO_4, and 2 mM KH_2PO_4, pH 7.4). After repeating this washing step one more time, the cells were pelleted by centrifugation for 3 min at 3,200 *g*, 4 °C, resuspended in 200 µl of 3% paraformaldehyde and incubated at room temperature for 15 minutes. The reaction was stopped by dilution of paraformaldehyde to a final concentration of 1% with PBS and the cells were pelleted by centrifugation for 3 min at 3,200 *g*, 4 °C. Cells were washed twice again with PBS and resuspended in PBS to reach an OD_{600} = 0.1; 50 µl of this cell suspension were laid on a coverslip pre-coated with poli-L-lysine (50 µl of poli-L-lysine, Sigma, were laid on a coverslip and dried overnight at room temperature) and incubated 15 minutes at room temperature. Then, each coverslip was deposited in a well of a 6-well culture plate and washed twice with 10 ml of PBS for 10 minutes with gentle stirring. The excess of buffer was wiped out and each coverslip was mounted face down in a slide with 15 µl of SlowFade Antifade Reagent (Thermo Fisher Scientific). The slide was stored in a dark box until the excess of mounting liquid dried, after which the slide and coverslip were sealed with nail polish. Samples were visualized in an Eclipse E800 fluorescence microscope (Nikon).

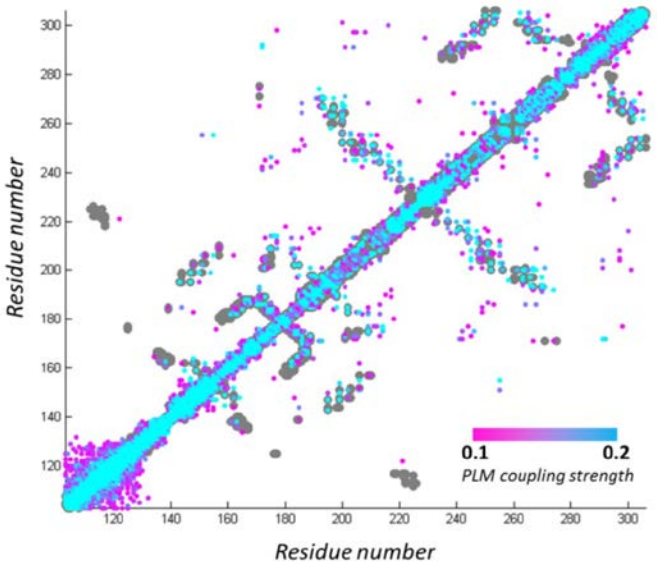

SUPPLEMENTARY FIGURE S1. Superposition of the contact map for ROBETTA Model 1 of the metalloprotease domain of MecR1 (gray) with the top 850 evolutionary couplings computed by EVCouplings (coupling strength increasing from pink to cyan). By disentangling different kinds of contributions to pairwise residue coevolution, the methods that analyze residue coevolution in a protein alignment are able to model protein structures ab initio with enough accuracy to assign the correct global fold provided that a sufficient number and quality of protein sequences is available [1]. By using the EVFold webserver [2] with no other data than BlaR1's or MecR1's metalloprotease domain sequences and standard parameters, we obtained models for the 130-300 regions whose backbone RMSDs were within 6 Å of the homology models, confirming the overall gluzincin fold. In particular, the contact map of model 1 returned by Robetta for MecR1 matches very well, as is, with the top 850 evolutionary couplings obtained by EVFold (coupling > 0.1446) which correspond to 5 times the length of the covered sequence segment (residue 130-300) as suggested in literature to reasonably estimate a model of a protein fold ab initio [2].

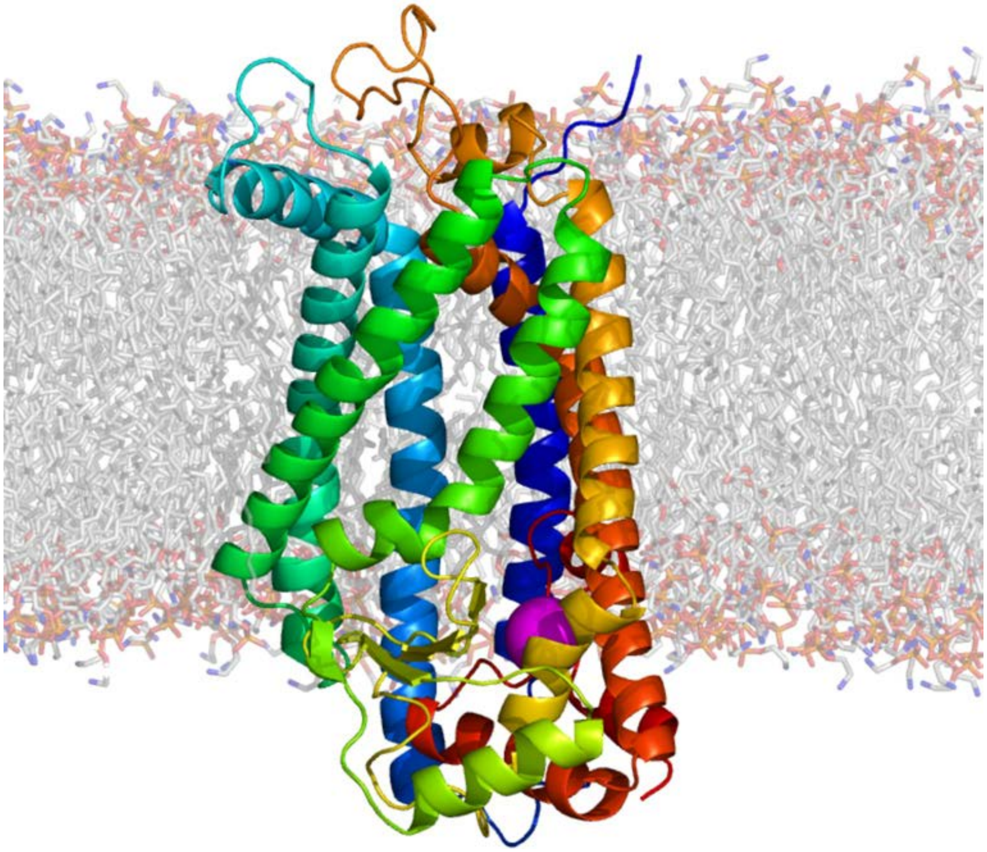

SUPPLEMENTARY FIGURE S2. The Ste24p protease from PDB ID 4IL3 relaxed in a POPE membrane through 1 microsecond of MARTINI coarse-grained MD simulation and 80 ns of atomistic (CHARMM36) MD.

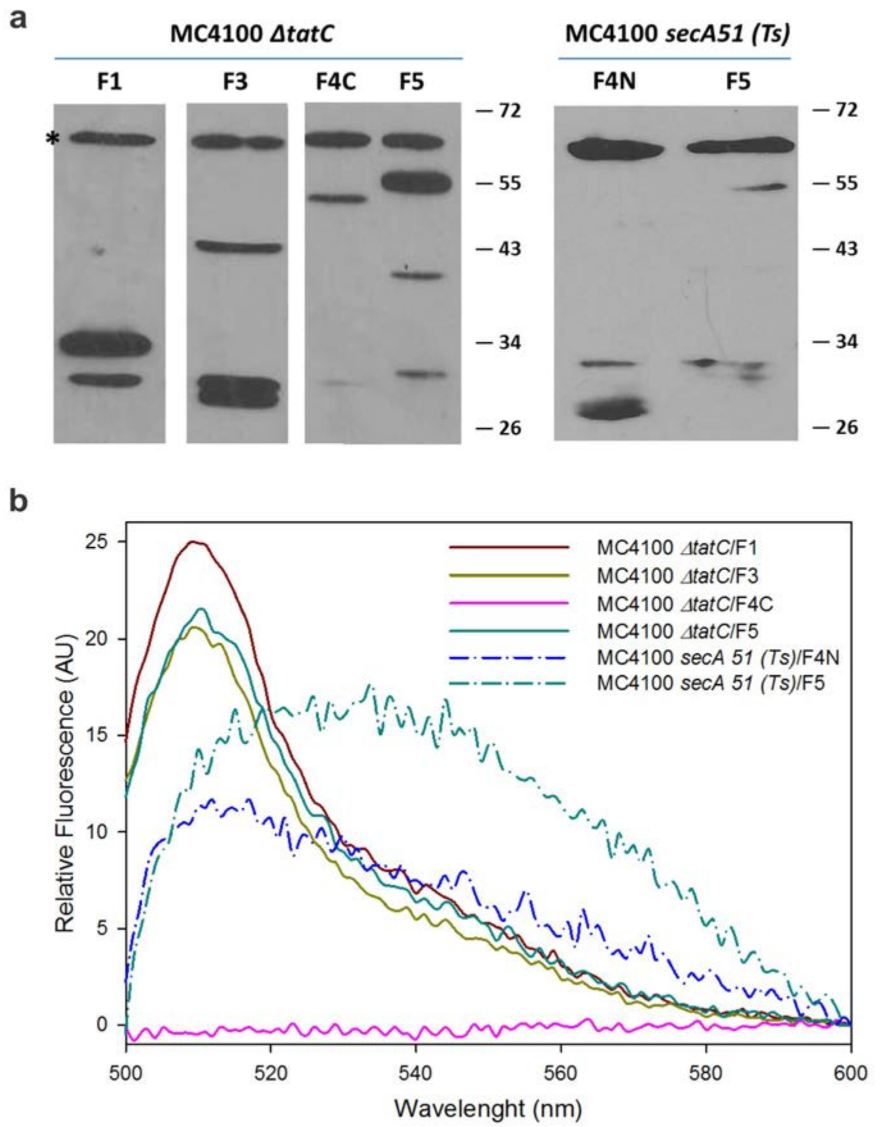

SUPPLEMENTARY FIGURE S3. Evaluation of the translocation system involved in the incorporation of MecR1 into the internal membrane of *E. coli* MC4100. The expression of the different MecR1-eGFP fusions was carried out in an *E. coli* MC4100 Tat system deletional mutant (Δ*tatC*) and in an *E. coli* MC4100 *secA* thermosensitive mutant (secA51 (Ts)). The level of recombinant protein expression in whole cell extracts was evaluated by Western blot ***(a)*** and by fluorescence spectroscopy of whole cells ***(b)***. Western blots were carried out using simultaneously a GFP specific primary antibody and a GroEL specific primary antibody with a Goat-anti-rabbit HRP-conjugated secondary antibody. Immunodetection of GroEL was used as a control of spheroplast integrity. The GroEL band is indicated with an asterisk. Full, uncut gel images are provided in Fig. S16. The GroEL band is indicated with an asterisk. The expression levels and fluorescence emission spectra of the fusion proteins in the Δ*tatC* mutant were similar to those observed in the WT strain. In contrast, most fusion proteins could not be expressed properly in the *secA51* thermosensitive mutant. These results corroborated that the Sec system was required for

proper expression and translocation to the membrane of MecR1 fusions, and validated the use of eGFP fusions for topology mapping of MecR1 in *E. coli*.

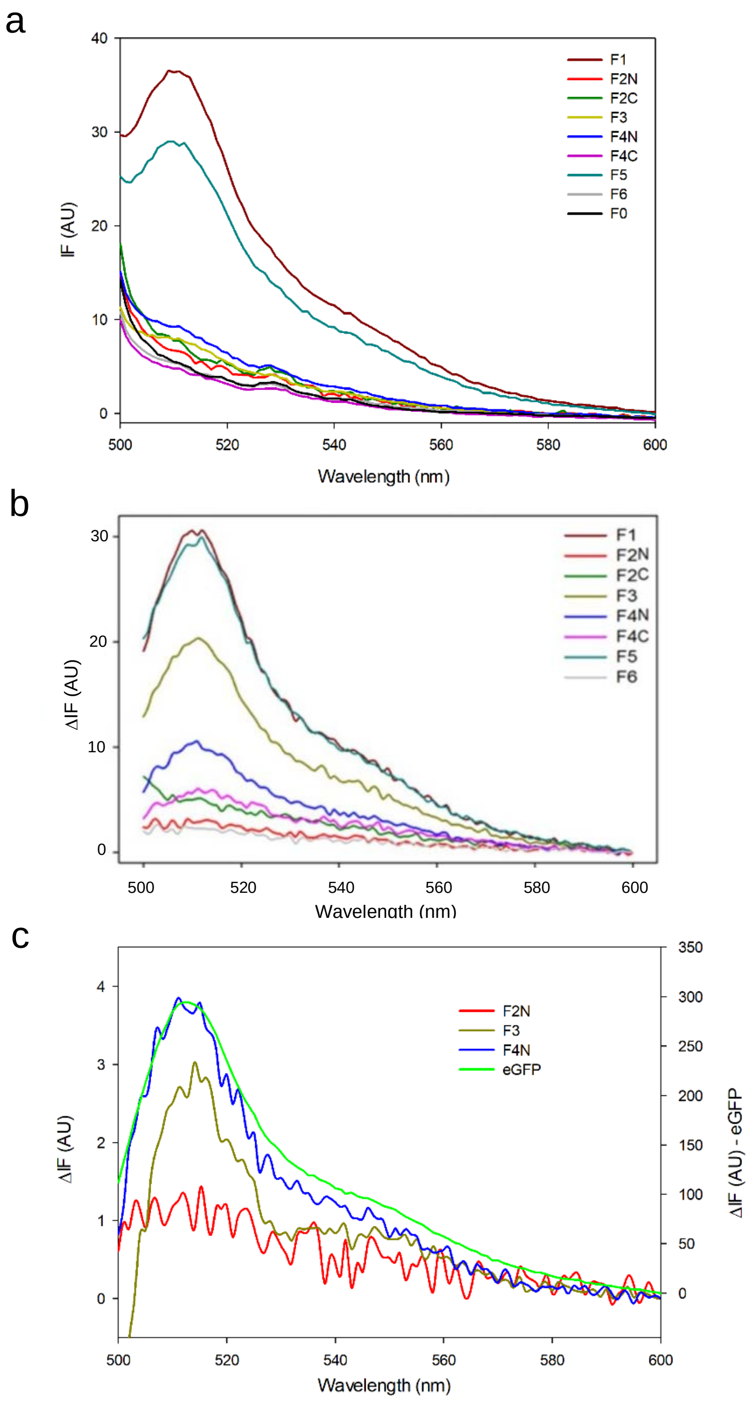

SUPPLEMENTARY FIGURE S4. Fluorescence Emission Spectra of the MecR1-eGFP fusions expressed in *E. coli*. ***(a)*** Fluorescence emission spectra of each MecR1-eGFP fusion in membrane preparations (F1 to F6) and of a membrane protein preparation from cells harboring the empty vector (Fo). The latter was subtracted from the spectra of F1-F6, to yield the spectra shown in Fig. 2.a. ***(b)*** Fluorescence emission spectra of each fusion in

whole cells, after subtraction of the spectra of the cells harboring the empty vector. The fluorescence intensity of F3 and F4N in whole cells was higher than the intensity observed for the proteins in membrane preparations, most likely due to degradation of the membrane protein, which would give rise to fluorescent eGFP in the cytoplasm of whole cells. F4C showed a fluorescence emission band typical of eGFP in whole cells (and accumulation of small amounts of eGFP due to proteolysis, Fig. S5) but presented no fluorescence in membrane preparations (Fig. 2.a and S4.a). ***(c)*** Comparison of the fluorescence emission spectra of eGFP (expressed in the cytoplasm, soluble) and that of the fusions (membrane protein preparations) displaying the lowest intensity. The spectra of F3 and F4N present maximum intensity at 512 nm, characteristic of eGFP.

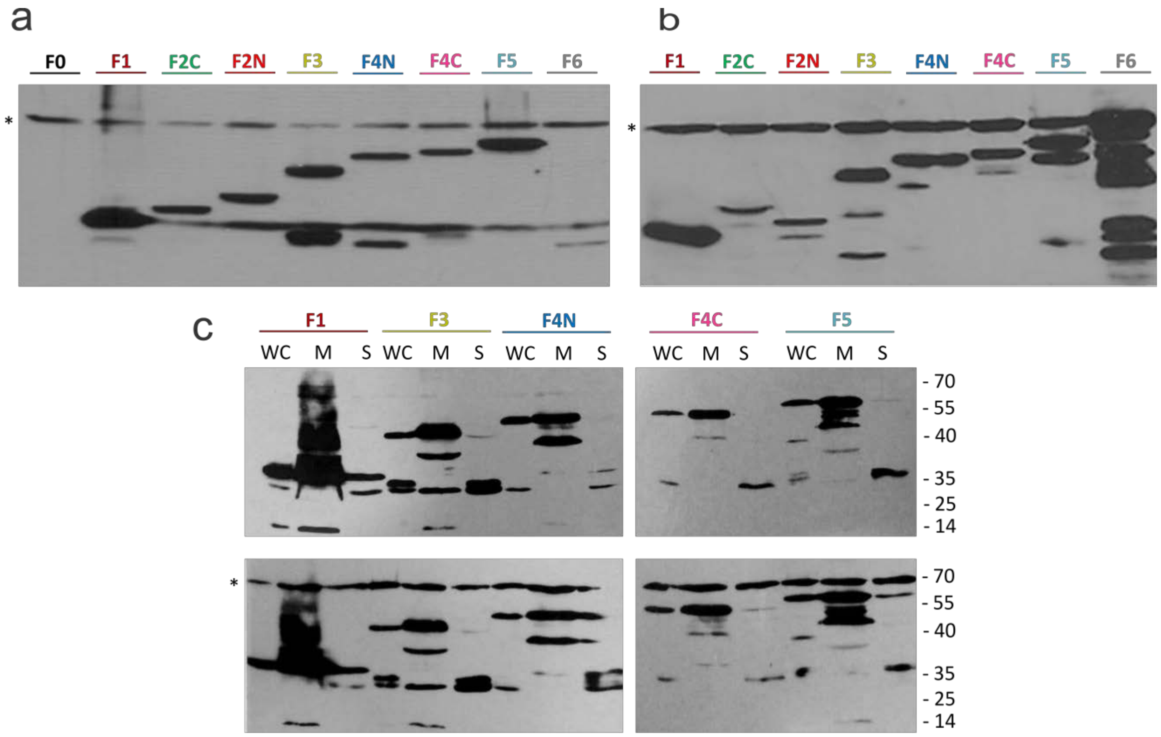

SUPPLEMENTARY FIGURE S5. Immunodetection of the MecR1-eGFP fusions expressed in *E. coli*. ***(a)*** Expression of the recombinant proteins MecR1-eGFP in whole cell extracts (*E. coli* MC4100 cells transformed with the corresponding gene cloned in pMBLe). The GroEL band is indicated with an asterisk and was used as a loading control. ***(b)*** Comparison of the level of expression of the different MecR1-eGFP proteins in membrane preparations. F1-F5: membranes from *E. coli* MC4100 cells transformed with the corresponding gene cloned in pMBLe. F6: *E. coli* BL21 Star (DE3) cells transformed with the corresponding gene cloned in pET24a(+). The cytoplasmic chaperone GroEL was found to partition to the membrane fraction upon expression of the recombinant membrane proteins MecR1-eGFP. Association of GroEL with membranes has been previously reported under stress conditions [3], and in our case might be due to stress imposed to the *E. coli* cells upon overexpression of membrane proteins. ***(c)*** Comparison of the proteins immunodetected in whole cells (WC), membrane protein preparations (M) and soluble protein fraction (S). The accumulation of proteolysis fragments in whole cells can be observed mostly for F3 and F4N, which then do not appear at such high levels in membrane preparations, but stay in the soluble protein fraction after ultracentrifugation. The fragments observed in the membrane preparations are most likely due to some degree of proteolysis after preparation of the membrane samples. In ***(a)*** and ***(b)***, Western blots were carried out using simultaneously a GFP specific primary antibody and a GroEL specific primary antibody. In ***(c)***, Western blots were carried out using a GFP specific primary antibody (top panel) or GFP snd GroEL specific primary antibodies, simultaneously (bottom panel).

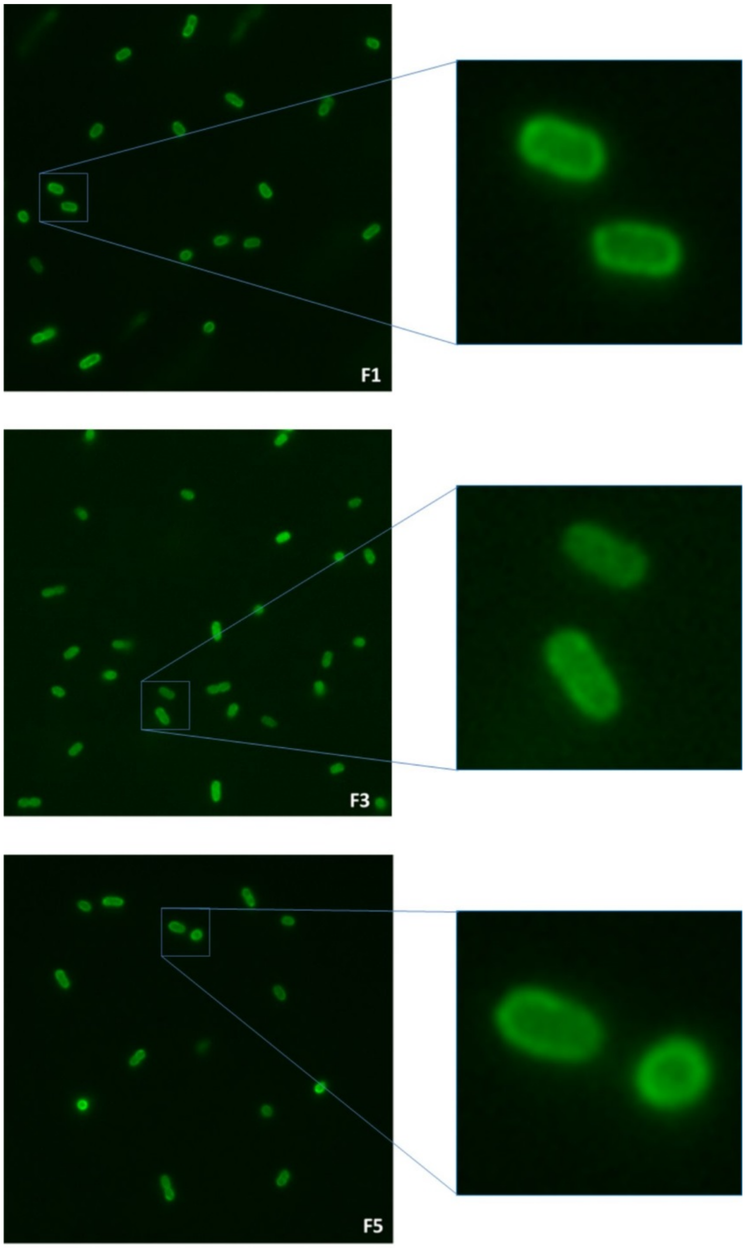

SUPPLEMENTARY FIGURE S6. Cellular localization of MecR1-eGFP fusions F1, F3 and F5 in *E. coli* MC4100 by fluorescence microscopy. Bacteria harboring the respective plasmid were induced and eGFP localization was determined by fluorescence microscopy. Fusions F2N, F2C, F4N, F4C and F6 were evaluated in the same manner, but the fluorescence level was not sufficiently high to be detected by this technique. F1 and F5 localize to the membrane in *E. coli*, while the fluorescence in the F3 version is distributed homogeneously in the cells. As shown in Fig. S3, F3 suffers proteolysis which releases eGFP to the cytoplasm and which might explain the observation of fluorescence in the cytoplasm by microscopy. The full-length version of F3 is subject to proteolysis even after membrane protein preparation in the presence of the protease inhibitor PMSF.

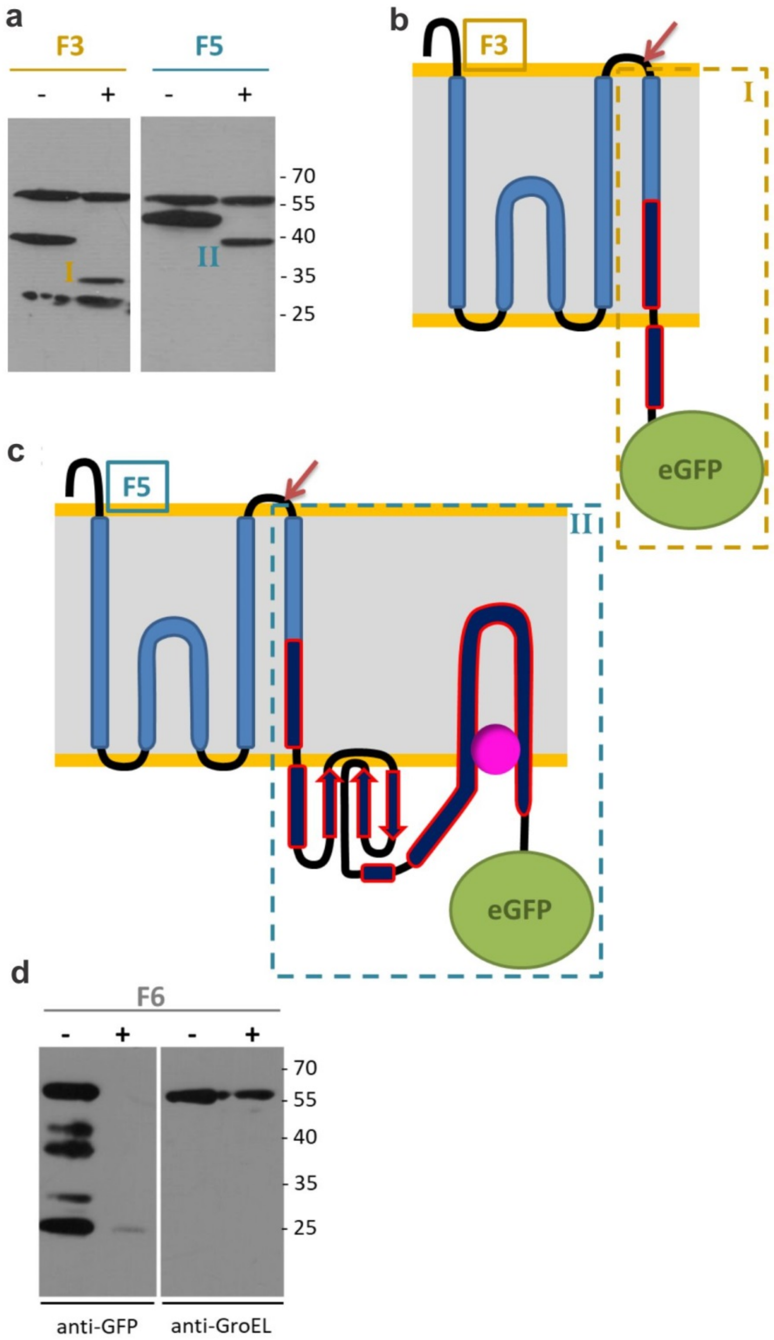

SUPPLEMENTARY FIGURE S7. Close-up of the Proteinase K susceptibility assay for MecR1_eGFP_F3 and _F5 truncated versions. ***(a)*** Proteinase sensitivity of F3 and F5 analyzed by Western blot using GFP and GroEL specific antibodies, simultaneously (extracted from Figure 2.*c*). The corresponding spheroplasts were incubated for 30 minutes at 0 °C without (-) or with (+) 100 µg/ml of Proteinase K. ***(b)*** and ***(c)*** Schemes for F3 and F5, respectively, if the constructs were inserted in membranes according to Robetta's

model (Model 1, Figure 1). Putative cleavage site of Proteinase K in Thr106 at the extracellular loop preceding TM3 is marked with a red arrow. Based on Robetta's model, the predicted molecular weight for the fragments resulting from Proteinase K digestion in fusions F3 and F5 is 33.8 kDa (I) and 46.4 kDa (II), respectively. ***(d)*** Proteinase K protection assay of the MecR1-eGFP-F6 fusion protein analyzed by Western blot using anti-GFP (left) and anti-GroEL (right) antibodies. The corresponding spheroplasts were incubated for 30 minutes at 0 °C without (-) or with (+) 100 μg/ml of proteinase K.

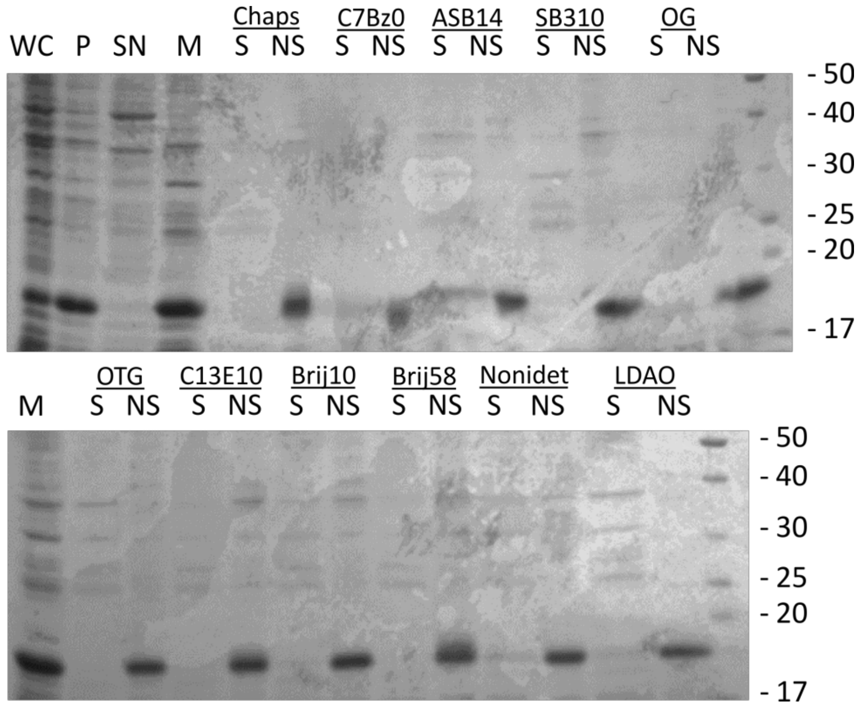

SUPPLEMENTARY FIGURE S8. Detergent screening to evaluate conditions to solubilize MecR1^{GLZ}.E205A from membranes. The fractions corresponding to solubilized proteins (S) and insoluble proteins (NS) are shown for each detergent tested. WC, whole cell extracts; P, pellet after centrifugation of sonicated cells; SN, supernatant after ultracentrifugation, M, membrane protein fraction after ultracentrifugation (fraction that was latter subjected to solubilization with different detergents).

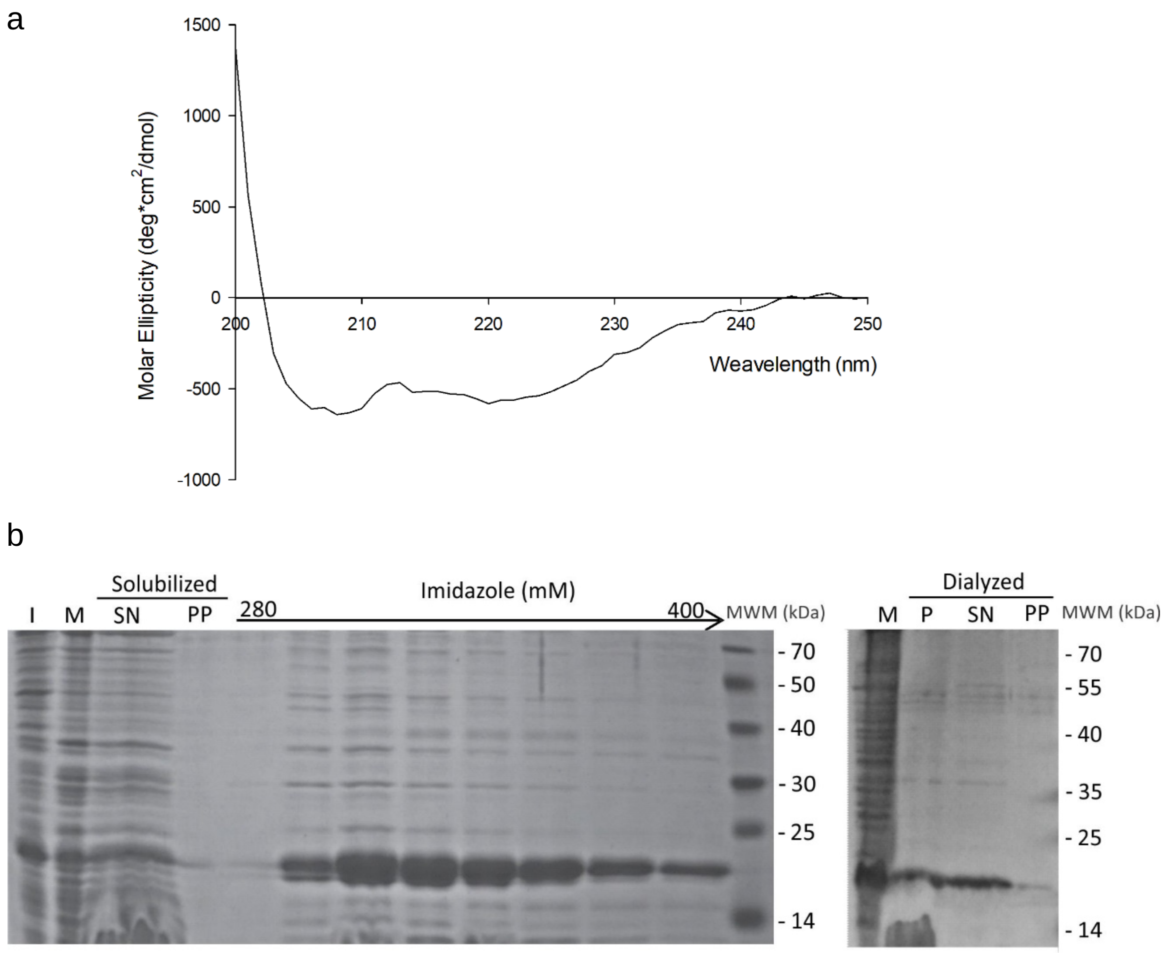

SUPPLEMENTARY FIGURE S9. Circular Dichroism spectrum of MecR1^{GLZ}.E205A. ***(a)*** Circular Dichroism spectrum of MecR1^{GLZ}.E205A in 10 mM NaH_2PO_4 pH 7.4 buffer, 300 mM NaCl, 6 mM KCl, 0.3% ASB-14, after subtraction of the spectrum of the buffer. Protein concentration was 4.5 µM. ***(b)*** SDS-PAGE gel (Coomassie stained) showing the different fractions of the purification of MecR1^{GLZ}.E205A and the final purity of the protein after purification using Ni-Sepharose and dialysis. Solubilization from membranes was carried out with addition of 2 volumes of 10 mM Na_2HPO_4; 1.8 mM NaH_2PO_4, 2.7 mM KCl, 2% ASB-14; 600 mM NaCl; 10% V/V glycerol, pH 7.4 buffer, for 16 h at 4 °C, with gentle agitation. Left: I, induced whole cell extract; M, membrane protein fraction; SN, soluble proteins fraction after solubilization of membrane proteins with ASB-14; PP, insoluble protein fraction after solubilization; lanes 5-12, elution fractions with Imidazole (280 to 400 mM). Right: M, membrane protein fraction; P, pre-dialysis protein sample; SN, soluble fraction after dialysis; PP, protein pellet after dialysis.

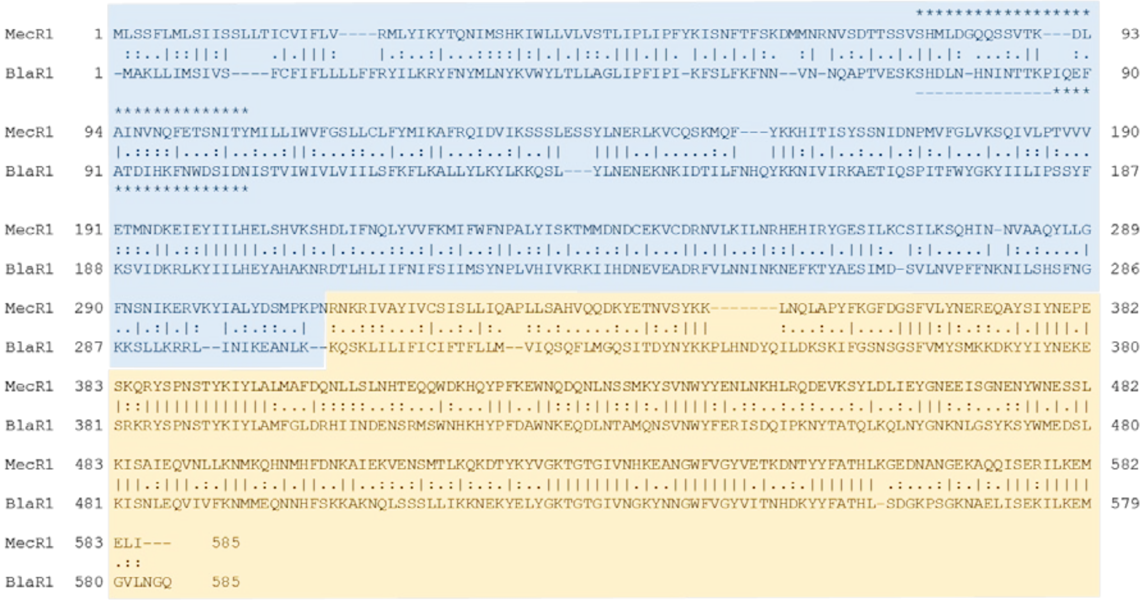

SUPPLEMENTARY FIGURE S10. Alignment of *S. aureus* BlaR1 and MecR1. Alignment was performed using EMBOSS' Needle webserver with standard settings. The asterisks under the BlaR1 sequence denote the residues that form the hydropathic helix observed to interact in the NMR studies by Frederick *et al.* [4], and the dashes that precede this segment complete the approximate definition of the L2 loop. The asterisks just above the MecR1 sequence correspond to the L2 loop as modeled in our Model 2, with its C-terminal half matching BlaR1's sequence the best. The section with light blue background corresponds to the Peptidase M56 domain as defined in PFAM (PF005569, dubbed "transmembrane", "metalloprotease" or "effector" domain) and the section with light orange background corresponds to the Transpeptidase domain as defined in PFAM (PF00905, the "sensor" domain).

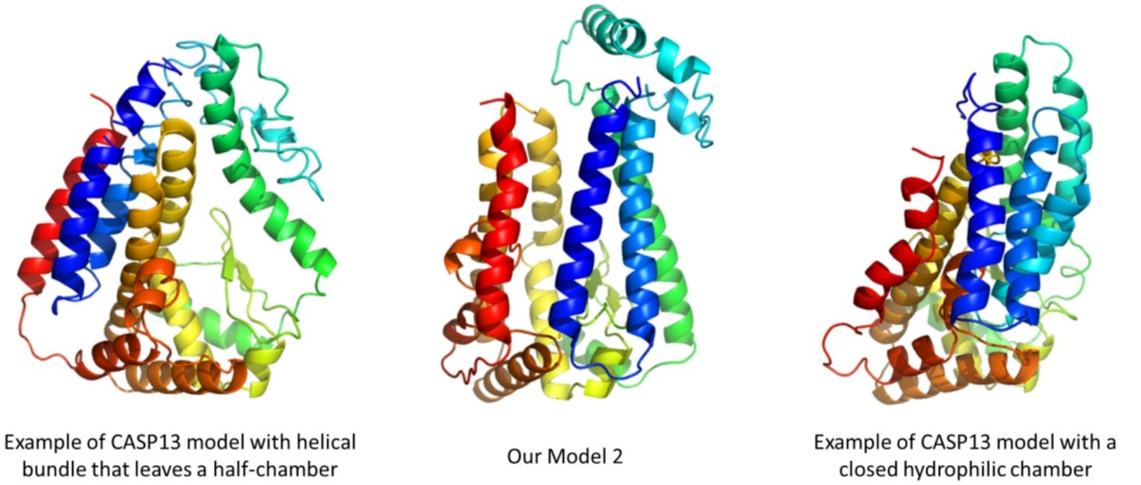

SUPPLEMENTARY FIGURE S11. Comparison to community-contributed models from CASP13. As a means to explore other plausible models of MecR1, we proposed its sequence as a target for CASP13, where 81 worldwide experts in molecular modeling (servers and human groups) provided five models each. None of the models captures the interaction observed experimentally between the sensor domain and L2, but this is not unexpected given its weak affinity. We therefore focused on region 1-333, i.e. the transmembrane domain. We inspected 259 models representatively different at 3Å, comparing them to our proposed model by using standard CASP scores. Encouragingly, models scoring in the top 20 were submitted by well-ranked CASP13 predictors (for example groups 089, 322, 145, 261, 197). All the models preserve a gluzincin core embedded in a transmembrane domain, but we observe two main groups of models depending on the packing of the transmembrane helices around it. In one group of models (left) the helices make a bundle on what would be the external side of the proposed hydrophilic chamber. As a result, this leaves its polar interior exposed directly to the hydrophobic portion of the membrane. In the other group of models (right) the helices arrange similarly to our model (middle), closing the hydrophilic chamber in the transmembrane domain; among these models, model 5 submitted by group 208 follows quite closely the transmembrane topology and structure we propose, with a Cα RMSD of 6.06 Å over 71% of the sequence essentially leaving out the L2 loop which is the hardest region to model, forming in this case two short transmembrane helices. There are two more interesting points to highlight. First, that the former configuration (i.e. with the TM helices not hiding the hydrophilic residues) cannot be discarded, as a dimeric form could effectively close the hydrophilic chamber avoiding exposure of its polar residues to the membrane. We unfortunately have no data about MecR1's oligomerization state. Second, that the L2 loop appears exposed to the "periplasmic" side in some models, but hidden inside the transmembrane domain (sometimes forming helices that close the chamber) on other models. Given our clear, double-checked observation that exposure of the L2 loop depends on the acylation state of the β-lactam binding domain, it is possible that the alternate conformations actually reflect a functional switch.

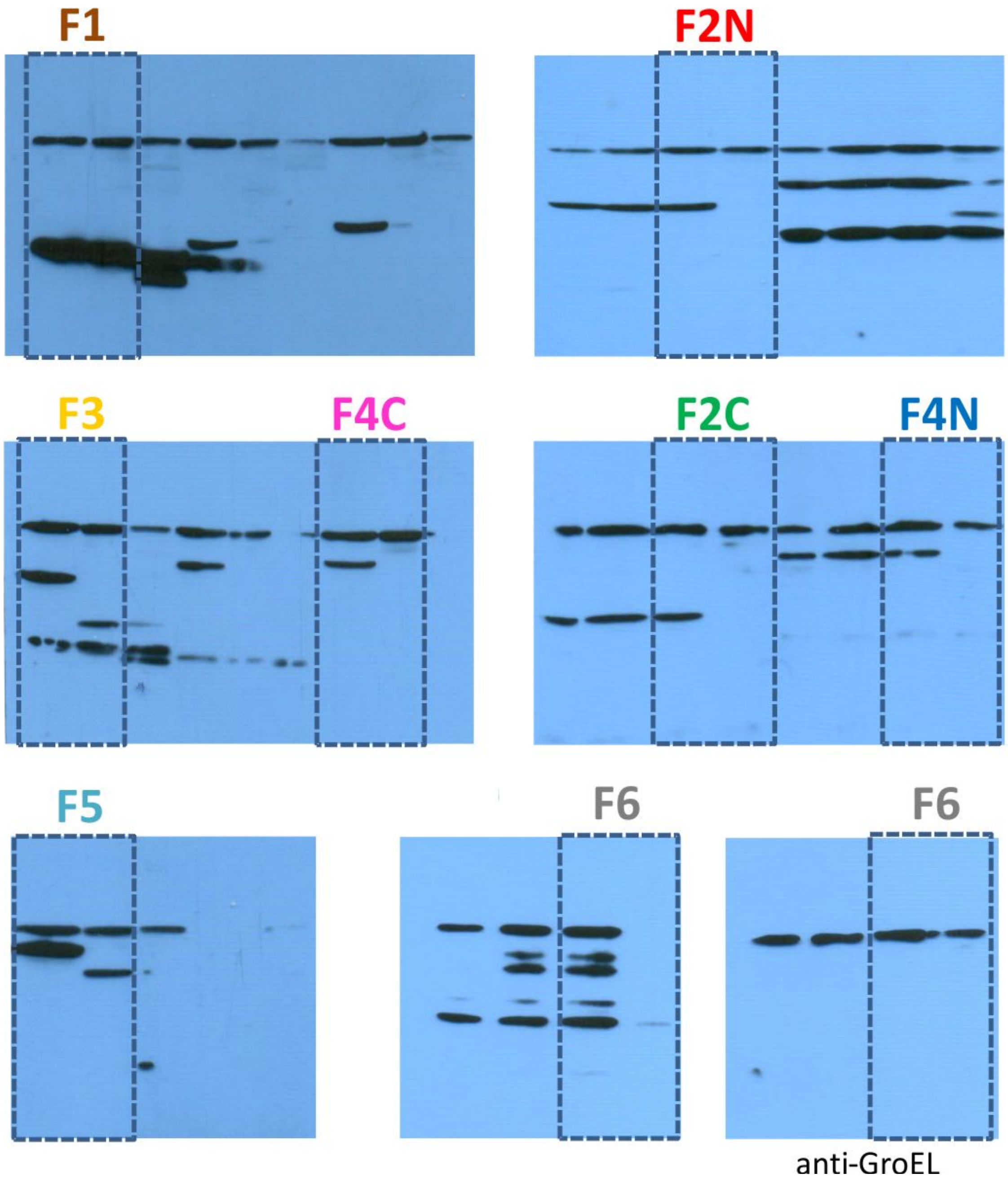

SUPPLEMENTARY FIGURE S12. Full, uncut gel images for Figure 2.b.

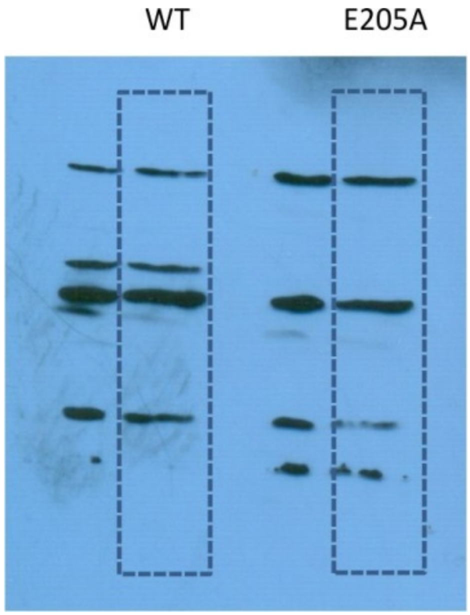

SUPPLEMENTARY FIGURE S13. Full, uncut gel images for Figure 3.a.

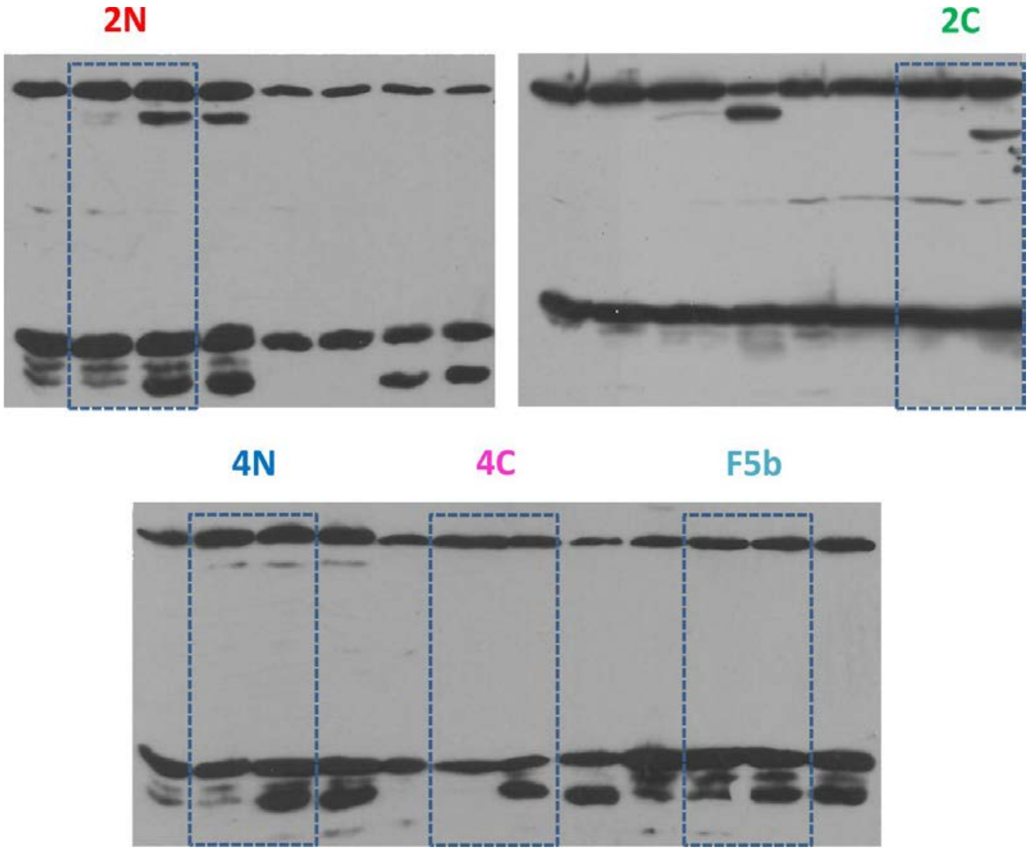

SUPPLEMENTARY FIGURE S14. Full, uncut gel images for Figure 3.b.

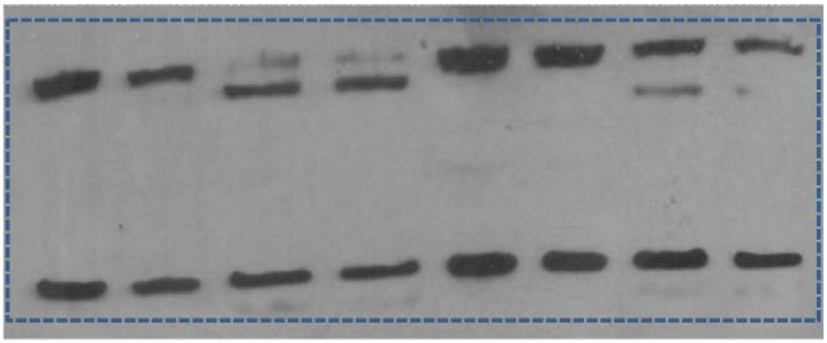

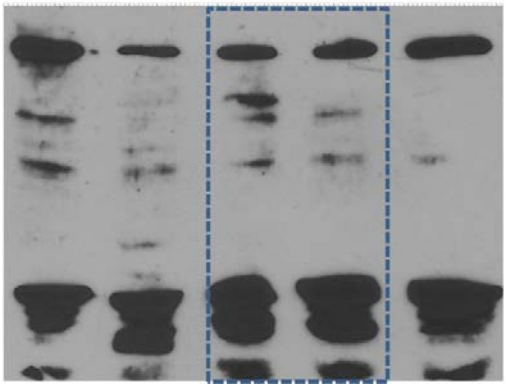

SUPPLEMENTARY FIGURE S15. Full, uncut gel images for Figure 3.d.

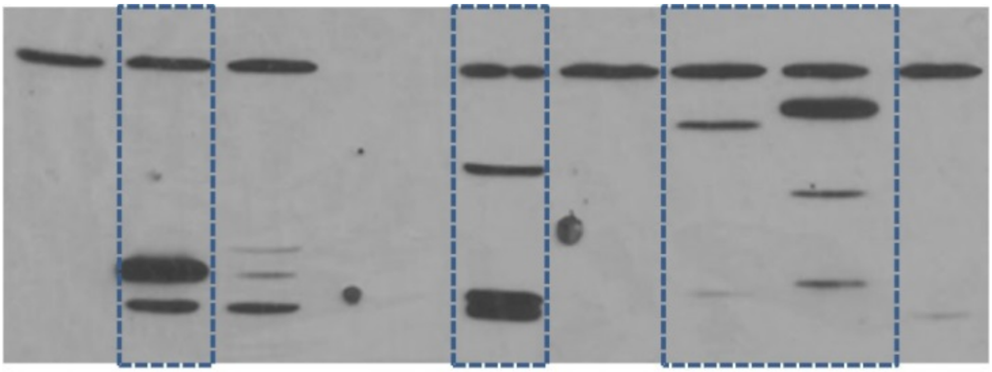

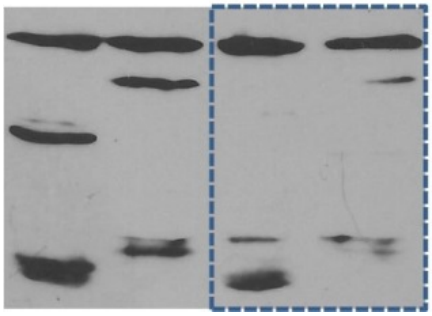

SUPPLEMENTARY FIGURE S16. Full, uncut gel images for Supplementary Figure S3.

SUPPLEMENTAL TABLE S1. Oligonucleotides used in this work.

Primer name	Primer Sequence (5'→3')
**oLL18**	GATATACATATGCTGAGTTCCTTCCTG
**oLL19**	GCGGATCCCGACATAATGTTCTGGG
**oLL20**	GCGGATCCGGTTTCAAACTGATTCACG
**oLL21**	CGCGGATCCGTGTTTTTTGTAGAATTGC
**oLL22**	CGCGGATCCGTCATGACTTTTCACG
**oLL23**	GCGGATCCAATGCTTTCGCCATAGCG
**oLL24**	GCGGATCCCTGCACATGTGCGGACAG
**oLL26**	CGCTCGAGAATCAGTTCCATTTCTTTCAGG
**oLL46**	GCGGATCCTTTATCGTTCATCGTTTCG
**oLL53**	CGAATACATCATCCTGCATGCACTGTCTCACG
**oLL54**	CGTGAGACAGTGCATGCAGGATGATGTATTCG
**oLL57**	GCGGATCCGTCAGACACATTACGGTTCATC
**oLL67**	GAAAACCTGTATTTTCAGGGCGGCAGCGACACCACGAGCTC
**oLL68**	GCTGCCGCCCTGAAAATACAGGTTTTCAGACACATTACGGTTCATC
**oLL69**	GAAAACCTGTATTTTCAGGGCGGCAGCACCTCTAACATCACGTATATG
**oLL70**	GCTGCCGCCCTGAAAATACAGGTTTTCTTCAAACTGATTCACGTTAATTG
**oLL71**	GAAAACCTGTATTTTCAGGGCGGCAGCAAAGAAATCGAATACATCATCC
**oLL72**	GCTGCCGCCCTGAAAATACAGGTTTTCATCGTTCATCGTTTCGAC
**oLL73**	GAAAACCTGTATTTTCAGGGCGGCAGCGACCTGATCTTTAATCAGCTG
**oLL74**	GCTGCCGCCCTGAAAATACAGGTTTTCATGACTTTTCACGTGAGAC
**oLL88**	GCGGATCCGCCCTGAAAATACAGGTTTTCGACAACCACGGTCGG
**oLL89**	GCGGATCCACGATGAACGATAAAGAAATCGAATACATC
**oLL90**	GCGGATCCGCCCTGAAAATACAGGTTTTCAATTTTCAGGACGTTACGATCAC
**oLL91**	GCGGATCCCTGAATCGTCATGAACACATCCG

REFERENCES

1 Abriata, L. A., Tamo, G. E., Monastyrskyy, B., Kryshtafovych, A. & Dal Peraro, M. Assessment of hard target modeling in CASP12 reveals an emerging role of alignment-based contact prediction methods. *Proteins* **86 Suppl 1**, 97-112, doi:10.1002/prot.25423 (2018).

2 Marks, D. S., Hopf, T. A. & Sander, C. Protein structure prediction from sequence variation. *Nat Biotechnol* **30**, 1072-1080, doi:10.1038/nbt.2419 (2012).

3 Torok, Z. *et al.* Evidence for a lipochaperonin: association of active protein-folding GroESL oligomers with lipids can stabilize membranes under heat shock conditions. *Proc Natl Acad Sci U S A* **94**, 2192-2197, doi:10.1073/pnas.94.6.2192 (1997).

4 Frederick, T. E., Wilson, B. D., Cha, J., Mobashery, S. & Peng, J. W. Revealing cell-surface intramolecular interactions in the BlaR1 protein of methicillin-resistant Staphylococcus aureus by NMR spectroscopy. *Biochemistry* **53**, 10-12, doi:10.1021/bi401552j (2014).
